# Early and Divergent Lipid Mediator Remodelling in Fast Versus Slow Skeletal Muscles of Female hSOD1^G93A^ Mice

**DOI:** 10.1002/jcsm.70357

**Published:** 2026-08-04

**Authors:** Sebastiaan Dalle, Kaat Vanderbeke, Thibaut Burg, Moniek Schouten, Wout Lauriks, Nicole Hersmus, Ludo Van Den Bosch, Katrien Koppo

**Affiliations:** ^1^ Exercise and Muscle Physiology Research Group, Department of Movement Sciences University of Leuven Leuven Belgium; ^2^ MOVANT Research Group, Department of Rehabilitation Sciences and Physiotherapy University of Antwerp Wilrijk Belgium; ^3^ Leuven Brain Institute, Department of Neurosciences University of Leuven Leuven Belgium; ^4^ Laboratory of Neurobiology, VIB Center for Neuroscience Leuven Belgium; ^5^ Department of Neurology University Hospitals Leuven Leuven Belgium

**Keywords:** ALS, cannabinoid receptor, endocannabinoid system, FAAH, neurodegeneration, SOD1

## Abstract

**Background:**

Skeletal muscle atrophy in amyotrophic lateral sclerosis (ALS) drives loss of muscle strength, function and quality of life in ALS patients. The endocannabinoid system (ECS) regulates muscle homeostasis via regenerative and metabolic processes, and although ECS alterations have been reported in ALS neural tissues, ECS remodelling within ALS skeletal muscle has never been studied. This study investigated temporal and muscle type–specific ECS changes in ALS.

**Methods:**

Female hSOD1^G93A^ transgenic mice and nontransgenic littermates were studied at presymptomatic and symptomatic ages (56–138 days of age; *n* = 7–8/group). Endocannabinoids, *N*‐acyl‐ethanolamine congeners and inflammatory lipid mediators were quantified using targeted LC–MS/MS in the tibialis anterior (TA) and soleus (SOL) muscles. ECS‐related enzymes and receptors were assessed by immunoblotting and integrated with transcriptomic analyses of skeletal muscle biopsies from ALS patients (*n* = 5/group; ~63 years). To evaluate therapeutic relevance, ALS mice were treated with the fatty acid amide hydrolase (FAAH) inhibitor URB937 or vehicle (*n* = 10–11/group), and survival, body weight, welfare and motor function were assessed longitudinally.

**Results:**

ALS caused severe atrophy in the predominantly fast‐twitch TA muscle (−76.5%; *p* < 0.01), while the slow‐twitch soleus was largely preserved (−14.4%; *p* < 0.01). Accordingly, the lipid perturbation due to ALS was more pronounced in the TA, reflected by extensive alterations in unsaturated fatty acids, hydroxy‐ and epoxy‐fatty acids (TA: 63% and SOL: 22% of lipid mediators different between ALS vs. NTG) and marked ECS remodelling, including elevated anandamide (+37.3%; *p* = 0.03) and multiple *N*‐acyl‐ethanolamine congeners (+76–102%; *p* < 0.05), reduced 2‐arachidonoylglycerol (−28%; *p* = 0.06), increased CB1 receptor expression (+93%; *p* < 0.01) and dynamic, age‐dependent regulation of FAAH (presymptomatic: −68%; *p* = 0.04, symptomatic: +21%; *p* = 0.02). In contrast, the SOL showed modest or opposite changes, consistent with its relative resistance to atrophy. Notably, ECS remodelling in the TA was already evident at presymptomatic age (e.g., CB1: +76%; *p* = 0.01) and the same ECS enzymes were affected in human ALS skeletal muscle transcriptomes (e.g., twofold decrease in FAAH; *p*_FDR_ = 0.010). Despite evidence for a therapeutic potential, chronic peripheral FAAH inhibition with URB937 did not improve weight loss, motor functions and survival of ALS mice (all *p* > 0.05).

**Conclusions:**

Muscle type–specific endocannabinoid system remodelling in ALS precedes overt neurological decline and might relate to degenerative features such as metabolic disturbance and inflammation. Although peripheral FAAH inhibition alone was insufficient to modify disease outcomes, these findings identify the endocannabinoid system as an integral component of ALS muscle pathology and support skeletal muscle lipid signalling as a potentially relevant early target for adjunctive therapeutic strategies.

## 1 Introduction

Skeletal muscle atrophy is an important driver of the pathophysiology of amyotrophic lateral sclerosis (ALS), a fatal neurodegenerative disorder characterized by progressive loss of motor neurons. ALS patients suffer from the loss of strength and motor function, and ultimately paralysis of the respiratory muscles, which results in premature death. ALS is driven by a complex combination of distinct genetic and environmental risk factors. Pathogenic variants in several genes (e.g., superoxide dismutase 1 [SOD1]) account for ~10% of the ALS cases, whereas the majority of the cases have no clear genetic cause (sporadic ALS) [1]. This complexity continues to pose a major challenge for the development of effective therapeutic strategies.

There is an increasing interest in the role of lipid metabolism as a central pathological hallmark of inherited and acquired neuropathies [2], such as ALS [3] and Charcot–Marie–Tooth disease [2]. Lipidomic analyses of human patient samples and animal models revealed remodelling of broad lipid classes (e.g., sphingolipids, glycerolipids, glycerophospholipids and sterols) in blood, cerebrospinal fluid and spinal cords of ALS patients and models [4]. In addition, phospholipids, sphingolipids and triglycerides were affected in skeletal muscle of a murine ALS model, both at presymptomatic and symptomatic ages [5]. In contrast to these broad lipid classes, the abundance and role of bioactive lipid mediators, such as eicosanoids and endocannabinoids, remains less understood in ALS muscle, although it has been suggested that they might be promising targets to slow disease progression (e.g., via neuroprotective, oxidative stress and inflammatory mechanisms) [4].

The endocannabinoid system (ECS) is one of the lipid mediator families that bridges metabolism, oxidative stress and inflammation [6]. The ECS consists of endocannabinoids such as 2‐arachidonoylglycerol (2‐AG), arachidonoyl ethanolamide or anandamide (AEA) and *N*‐acyl‐ethanolamine congeners such as *N*‐palmitoylethanolamine (PEA), *N*‐stearoylethanolamine (SEA) and *N*‐oleoylethanolamine (OEA). Their targets include G protein–coupled receptors (GPRs), including cannabinoid receptor 1 (CB1), CB2, GPR55 and GPR119, transcription factors such as peroxisome proliferator‐activated receptors, nuclear receptors and ion channels [7]. In addition, endocannabinoids and their congeners can be enzymatically metabolized via lipoxygenases, cyclooxygenases and cytochromes P450 to form inflammatory lipid mediators with distinct biological functions. Data from ALS patients and murine models indicate that the circulating and spinal cord endocannabinoid levels are increased [8] and positively associate with the disease status and the disease duration [9]. These increases are a (neuro)protective compensation mechanism to exert anti‐inflammatory, antioxidative stress, analgesic and antiexcitotoxicity effects [10]. There are also positive indications regarding the use of (phyto)cannabinoids such as THC and CBD for therapeutic purposes such as (perceived) relief of muscle spasticity and pain in ALS patients, but large, qualitative studies are still needed [11]. In addition, the role of the ECS in ALS disease progression remains incompletely understood.

Whereas most ALS research focuses on the pathophysiology of motor neuron degeneration, the role of skeletal muscle, which bidirectionally communicates with motor neurons via the neuromuscular junction, is pivotal [12]. Neuromuscular and muscular changes, such as defective mitochondrial dynamics, can precede deficits in motor function and motor neuron loss [13, 14], which makes skeletal muscle an important target in the ALS pathophysiology. Whereas ECS physiology has already been studied in the neural ALS pathophysiology, it has never been studied whether ALS skeletal muscle undergoes similar ECS remodelling as observed in other preclinical and clinical models of muscle degeneration, such as sarcopenia [15], Duchenne muscular dystrophy [16], muscle injury [17] and cancer cachexia [18]. Interestingly, a growing body of evidence indicates that ECS targeting via genetic or pharmacological approaches modulates muscular processes such as stem cell growth, inflammation, metabolism and regeneration [16, 17, 19, 20, 21], which are all affected in ALS muscle.

The present study aims to uncover whether the skeletal muscle ECS underlies ALS‐related muscle atrophy. Lipid mediator profiling is performed via liquid chromatography–tandem mass spectrometry (LC–MS/MS)–based targeted mediator lipidomic analysis in the tibialis anterior muscle (TA) and the soleus muscle (SOL) of female mice overexpressing the human mutant SOD1^G93A^ (ALS) and female nontransgenic littermate controls (NTG) at different ages. Whereas the TA (primarily fast‐twitch fibres) is highly affected by ALS, the SOL (more slow‐twitch fibres) weight and molecular profile are relatively well preserved in ALS mice compared with NTGs [22]. Therefore, the present study provides a unique approach that allows for distinguishing between differences that can be ascribed to the model itself (i.e., when comparing ALS with NTG) and to the muscle atrophy within the model (i.e., when comparing TA and SOL in ALS mice).

## 2 Methods

### 2.1 Mice

Female mice overexpressing human mutant SOD1 (B6SJL‐Tg [SOD1*G93A] 1Gur/J; Stock Number: 002726; a model of ALS) and nontransgenic littermate controls were bred at the KU Leuven Laboratory Animal Center. Mice were housed in a controlled 12‐h light/12‐h dark cycle at 21°C–22°C and were fed ad libitum on a standard diet (Ssniff‐Spezialdiäten GmbH, Soest, Germany) and water.

### 2.2 Study Design—Experiment 1

**FIGURE 1:**
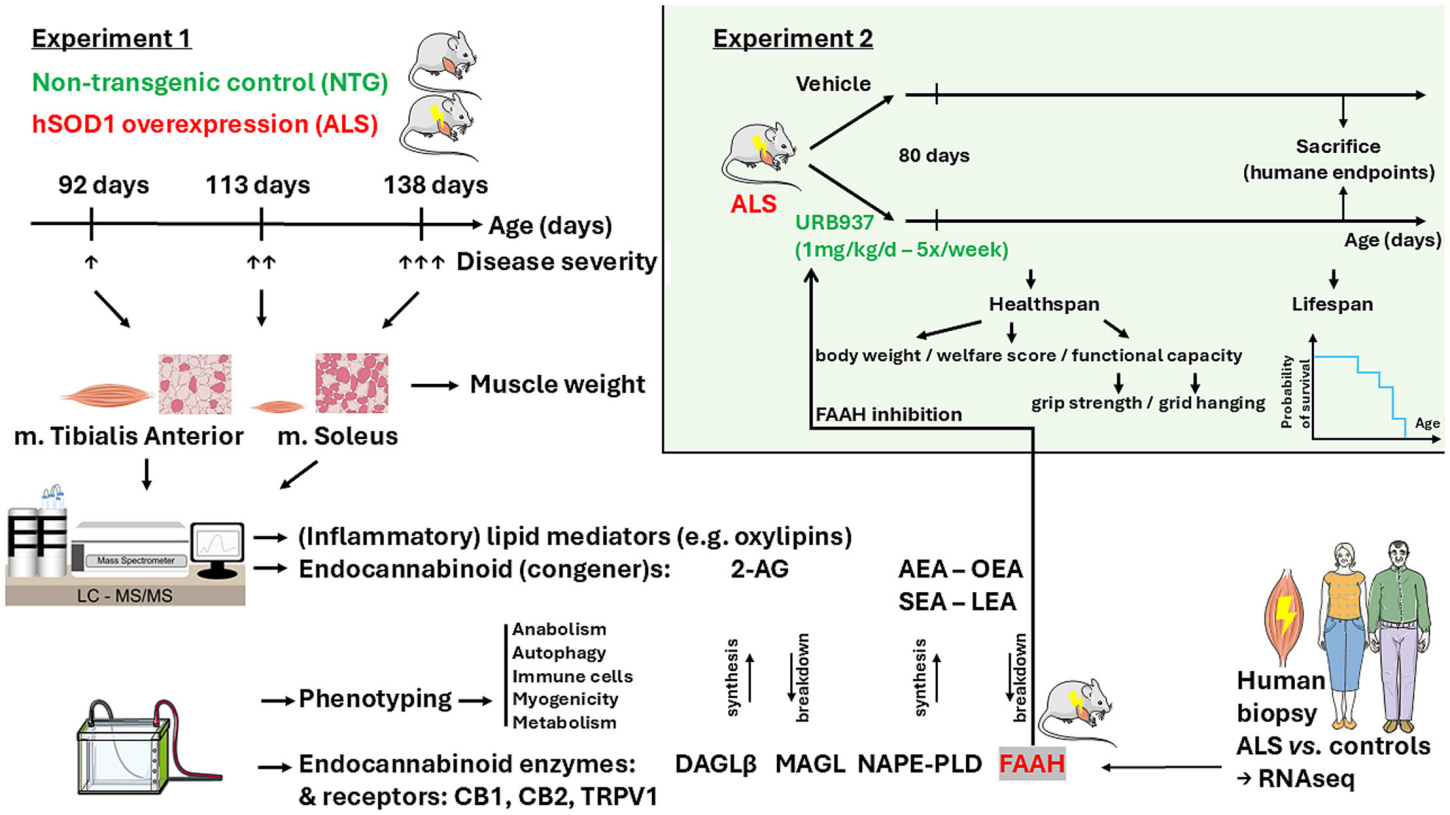
A schematic overview of the study protocol of experiment 1 and experiment 2 is provided. Shortly, in experiment 1, female mice overexpressing human mutant SOD1 (model of amyotrophic lateral sclerosis or ALS) and nontransgenic littermate controls were sacrificed at 92, 113 and 138 days of age to study endocannabinoid (congener) levels, endocannabinoid enzymes and receptors in the predominantly fast‐twitch tibialis anterior and the predominantly slow‐twitch soleus muscle. Murine endocannabinoid enzyme remodelling was paralleled in humans via re‐analyses of RNA sequencing data obtained in skeletal muscle of ALS patients and healthy controls. In experiment 2 (green background), ALS mice were treated with vehicle or the FAAH inhibitor URB937 (1 mg/kg/d; five times per week) starting from the age of 80 days until humane endpoints were reached. Measures of health and lifespan were assessed.

Mice were sacrificed via cervical dislocation at three ages, that is, 92 days (*n* = 8 ALS and *n* = 8 NTG), 113 days (*n* = 7 ALS and *n* = 8 NTG) and 138 days of age (*n* = 8 ALS and *n* = 8 NTG) (Figure 1). These ages represent different levels of severity in ALS pathology. Functional characteristics such as body weight, grip strength performance and welfare score at these three ages are presented in Table S1 including mice from study 2 (different mice, same colon). Randomization of mice into conditions was based on their genotype and age. Upon sacrifice, hindlimb muscles were harvested, snap‐frozen in liquid nitrogen, weighed and stored at −80°C, and the TA and SOL were further analysed. The experiment was approved by the KU Leuven Animal Ethics Committee (M022/2023), and all methods were performed in accordance with the relevant guidelines and regulations, including the ARRIVE guidelines.

### 2.3 Power calculation—Experiment 1

Skeletal muscle degeneration, that is, acute injury, increases CB1 expression compared to noninjured muscle [17] (*η* ^2^ = 0.45; *f* = 0.90) and with *α* = 0.001 (to account for multiple testing 0.05/50 including functional and molecular outcomes), power (1‐β) = 0.95, 6 groups, numerator df = 2, *n* = 7/group should be included.

### 2.4 Study design—Experiment 2

From the age of 80 days (onset of disease symptoms), female mice overexpressing human mutant SOD1 (B6SJL‐Tg [SOD1*G93A] 1Gur/J; Stock Number: 002726) were randomized based on body weight to be intraperitoneally injected (6 mL/kg body weight) five times per week with either vehicle (97.9% saline; 1.7% DMSO; 0.4% tween‐80; *n* = 10) or with URB937 (1 mg/kg/d) dissolved in vehicle (*n* = 11) (Figure 1). URB937 is a peripherally restricted inhibitor of the endocannabinoid‐degrading enzyme fatty acid amide hydrolase (FAAH). A dose of 1 mg/kg decreases FAAH activity with ~50% in peripheral but not central tissue, resulting in increased levels of AEA (~ninefold), PEA (~fivefold) and OEA (~fourfold) in peripheral but not central tissues [23, 24]. URB937 was stored at −20°C in a stock solution of 80% dimethylsulfoxide (DMSO) and 20% tween‐80. The stock solution was thawed and saline was added freshly before intraperitoneal injection. The experiment was approved by the KU Leuven Animal Ethics Committee (BW‐034/2025), and all methods were performed in accordance with the relevant guidelines and regulations, including the ARRIVE guidelines.

### 2.5 Power Calculation—Experiment 2

FAAH genetic ablation improves muscle force (ES = 4.8) in hSOD1^G93A^ mice [25], and with *α* = 0.01 (to account for multiple testing 0.05/5 including body weight, survival, motor capacity testing and welfare scoring), power (1‐β) = 0.95, this would require only *n* = 3/condition, which is inappropriate for survival analyses. Therefore, in parallel with a previous work that assessed survival in hSOD1 mice following endocannabinoid modulation [25], *n* = 10–11/condition was included.

Protein extraction—Experiment 1: as previously described [15, 17, 18] (see Supporting Information).

Western blot analysis—Experiment 1: as previously described [15, 17, 18] (see Supporting Information).

### 2.6 Lipid Extraction—Experiment 1

The extraction protocol was adapted with modifications from Dumlao et al. [26] and has been previously applied in skeletal muscle tissue [17, 18]. TA and SOL tissue samples (~15 mg) were mixed with methanol containing 100 pg deuterated internal standards and 10 μL antioxidant mix (100 μM indomethacin, 0.2 mg/mL butylated hydroxytoluene [BHT], 100 μM trans‐4‐(‐4‐(3‐adamantan‐1‐yl‐ureido9‐cyclohexyloxy)‐benzoic acid [t‐AUCB] in MeOH) and were homogenized using a Precellys system at 4°C. The homogenized samples were stored at −80°C for 30 min and then centrifuged at 16000 g for 10 min at 4°C. The supernatant was transferred to a new tube and diluted with water to achieve a methanol percentage < 10%. The remaining pellet was redissolved in TE buffer for DNA concentration determination (Hoechst assay). Lipids were extracted using Strata‐X 33 μm Polymeric Reversed Phase extraction columns (Phenomenex, 8B‐S100‐ECH) as instructed by the manufacturer. Briefly, the columns were preconditioned with 3 mL of methanol, followed by 3 mL of water. The sample (containing < 10% methanol) was eluted dropwise, followed by a wash step with 3 mL of 10% methanol and elution with 100% methanol into a glass collection tube containing 6 μL of a 30% glycerol in methanol solution. Samples were evaporated in a vacuum centrifuge, redissolved in a 1:1 solution of water/methanol and transferred to an LC vial.

### 2.7 LC–MS/MS–Based Lipidomics—Experiment 1

Lipid species were analysed by liquid chromatography electrospray ionization tandem mass spectrometry on a Nexera X2 UHPLC system (Shimadzu) coupled with a hybrid triple quadrupole/linear ion trap mass spectrometer (QTRAP 6500+ system; SCIEX). Chromatographic separation was performed on a Polar C18 column (2.6 μm, 3.0 × 100 mm; Phenomenex) maintained at 50°C, using mobile phase A (0.1% formic acid in water) and mobile phase B (0.1% formic acid in methanol) in the following gradient: 0–2 min: 45% B; 2–16.5 min: 45% B → 80% B; 16.5–18.5 min: 98% B; 18.5–20.5 min: 10% B; all at a flow rate of 0.5 mL/min. The instrument parameters were as follows: curtain gas = 30 psi; collision gas = 8 a.u. (medium); ion spray voltage = 5200 V and −4200 V; temperature = 520°C; ion source gas 1 = 50 psi; ion source gas 2 = 70 psi. The endocannabinoids, leukotrienes and related mono‐ and polyhydroxylated tetraene metabolites (MTCR and PTCR) were detected in positive ion mode and all other compounds were detected in negative ion mode. Lipid abundance was normalized to tissue DNA content. MRM transitions were sourced from Stassburg et al. [27]. Lipidomic analyses were performed in *n* = 5 in NTG and ALS mice, respectively, and only at the age of 113 days.

### 2.8 Behavioural, Health and Functional Testing—Experiment 2

From the age of 80 days, behaviour and health were scored five times per week and the functional capacity was assessed three times in 10 days. Behavioural and health testing included scoring (score between 0 and 4 for every parameter) by the same researcher for (1) appearance, (2) activity level, (3) eyes, (4) pain and distress behaviour, (5) breathing, (6) neurological scoring and (7) body weight loss over a 7‐day interval. A detailed scoresheet of each parameter is added in the Supporting Information. Functional motor capacity testing included five grip strength attempts and grid hanging (maximal three attempts if mice failed to reach a 1‐min hang). Grip strength measurement was ceased when mice were unable to pull the grid, and grip strength failure was determined as the first time point at which the average of the three best attempts was below 50 g. Grid hanging failure was assessed as the second consecutive day on which mice failed to hang for 1 min (six attempts not reaching a 1‐min hang).

### 2.9 Humane Endpoints—Experiment 2

Animals were killed if one of the humane endpoints was reached: (1) a summed score of 14 or higher (max score of 28) on the behavioural and health test (assessed five times per week), (2) a score of 4 for any of the following: appearance, activity level, pain and distress behaviour, neurological scoring, body weight loss (assessed five times per week) and (3) probably not alive during a following observation according to the researcher's judgement (assessed five times per week).

### 2.10 Human Transcriptomic Analyses

To parallel endocannabinoid‐related muscle remodelling of the murine ALS model towards human skeletal muscle, we made use of ribonucleic acid (RNA) sequencing data deposited in GSE215424 [28]. Skeletal muscle biopsies of five healthy (60.8 ± 9.6 years; 1 female; three biceps brachii and two deltoid muscles) and five ALS patients (64.4 ± 14.1 years; three females; four biceps brachii and 1 deltoid muscle) were obtained from the University of Alabama at Birmingham Neuromuscular Division, and ALS was diagnosed based on the revised E1 Escorial criteria.

The RNA sequencing procedure and gene count data handling are described in the Supporting Information.

### 2.11 Data Analyses—Experiments 1 and 2

Results are presented as mean and individual data points (Experiments 1 and 2) or standard error of measurement (experiment 2). Comparison of conditions was done with two‐way analysis of variance (Experiment 1: 2 genotypes × 3 ages; Experiment 2: time × 2 treatments) and Bonferroni‐corrected post hoc comparisons in case of a significant interaction effect, or with Student's *t*‐test (normally distributed data) or Mann–Whitney *U* test (not normally distributed data). Normality was tested via the Shapiro–Wilk test. Survival curves (survival, grid hanging failure and grip strength failure) were analysed via the Gehan–Breslow–Wilcoxon test. Significance level was set at *p* < 0.05. Statistics were performed using GraphPad Prism 8 (GraphPad Software Inc., San Diego, CA, USA). Power calculations were performed with G*Power 3.1.9.7.

For the human transcriptomic analyses, low‐count genes (below 10 counts) were prefiltered and the read counts of RNA transcripts were normalized (size factor normalization). Differential abundance between healthy and ALS individuals was assessed using the DESeq2 package (Version 1.51.3) pipeline in R (Version 4.2.2). Statistical testing was performed using the Wald test. RNAs were defined as differentially regulated with an absolute log2 fold change > 0.6 and Benjamini–Hochberg adjusted *p* value < 0.05.

## 3 Results

### 3.1 Fast‐Twitch Muscles Are Vulnerable Compared to the Relatively Well Preserved Slow‐Twitch Soleus Muscle in ALS Mice

**FIGURE 2:**
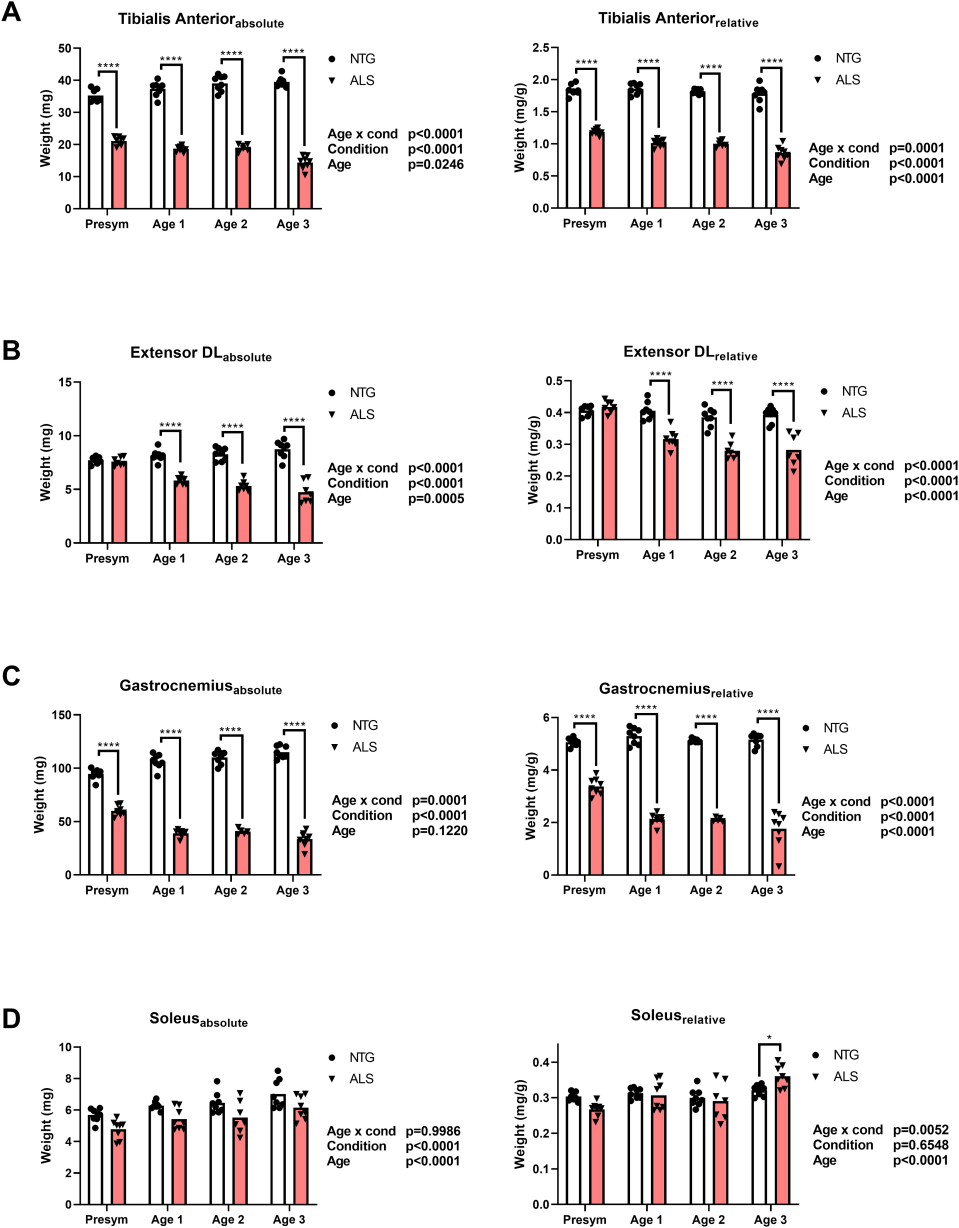
The effect of the hSOD1 genotype and age on the absolute and relative (relative to body weight) weight of the tibialis anterior muscle (panel A), the extensor digitorum longus muscle (extensor DL; panel B), the gastrocnemius muscle (panel C) and the soleus muscle (panel D). The individual (dots and triangles) and average (bar) values of nontransgenic control mice (NTG; dots) and hSOD1‐transgene mice (ALS; triangles) are presented. Age 1, 2 and 3 refer to an age of 92, 113 and 138 days, respectively. *p* values of two‐way analysis of variance, and Bonferroni‐corrected post hoc analyses are presented (*n* = 7–8/group except for *n* = 5 in gastrocnemius of ALS age 2).

ALS induced a pronounced absolute and relative muscle (to bodyweight) loss in primarily fast‐twitch muscle types such as the TA (abs. –76.5%; rel. –62.4%), extensor digitorum longus (EDL) (abs. –45.4%; rel. –29.7%) and gastrocnemius (abs. –98.1%; rel. –89.0%) muscle (Figure 2A–C). In SOL, ALS still had an effect on the absolute weight (−14.4%), but not the relative weight (+2.1%; Figure 2D). The absolute muscle weights of all primarily fast‐twitch muscle types exhibited a significant interaction effect, representing slightly higher muscle weights with advancing age in NTG (+7.0 ± 1.1%), whereas lower muscle weights with advancing age were observed in ALS (−18.4% ± 4.5%; Figure 2A,B,D).

**FIGURE 3:**
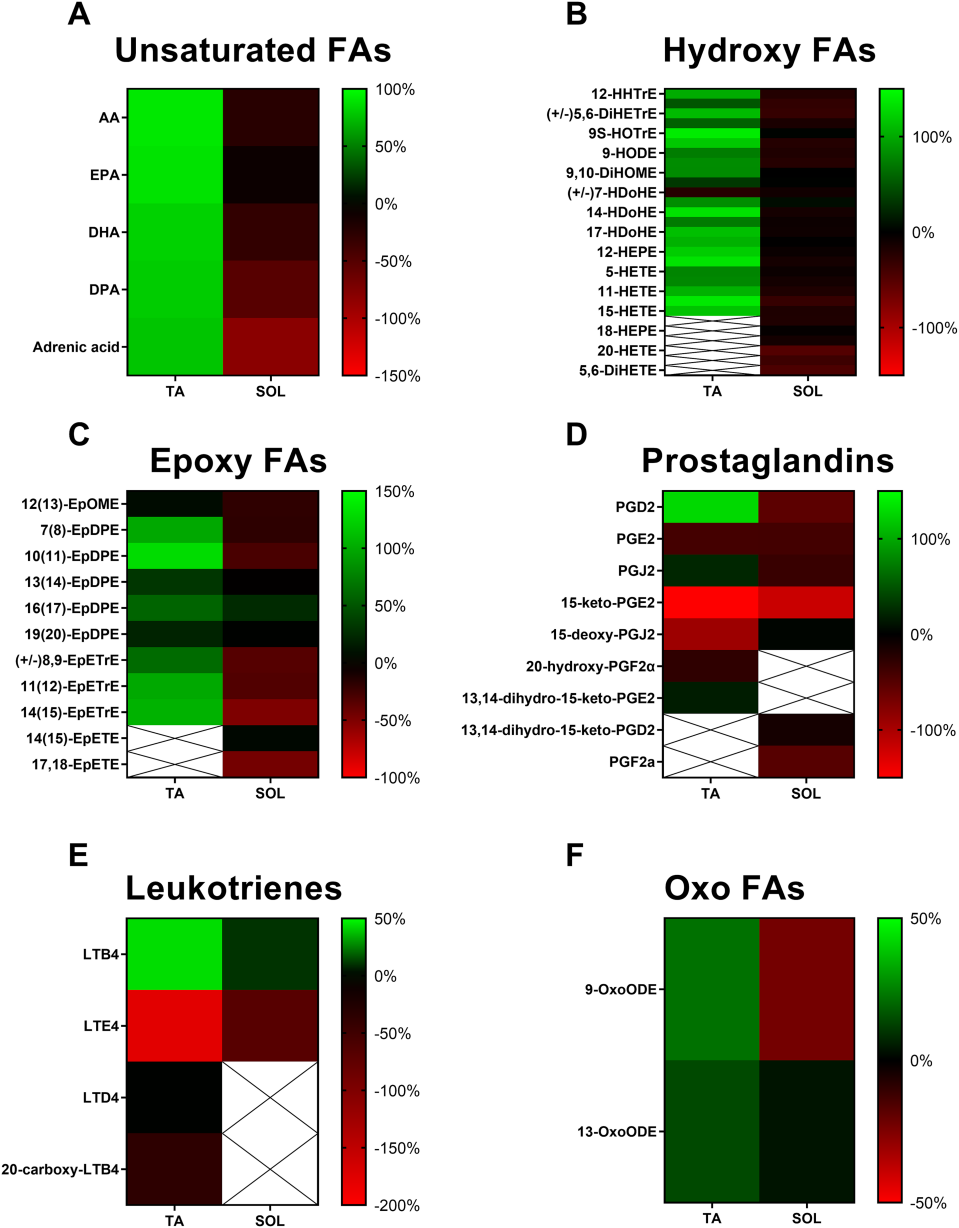
The effect of hSOD1 genotype on the inflammatory lipid mediator abundance in the tibialis anterior (TA) and soleus muscle (SOL). Lipid mediators include the unsaturated fatty acids (FAs; panel A) arachidonic acid (AA), EPA, DHA, DPA and adrenic acid. Hydroxy fatty acids (FAs; panel B) include HHTrEs, DiHETrEs, HOTrEs, HODE, DiHOME, HDoHE, HEPEs, HETEs, DiHETE. Epoxy fatty acids (FAs; panel C) include EpOME, EpDPEs, EpETrEs, and EpETEs. Other lipid mediators are prostaglandins (PGs; panel D), leukotrienes (LTs; panel E) and oxo fatty acids (FAs; panel F), that is, oxoODEs. The heat maps show the %‐difference in lipid mediator muscle abundance between the hSOD1‐transgene and nontransgenic control mice, for the TA and SOL (*n* = 5/group).

**FIGURE 4:**
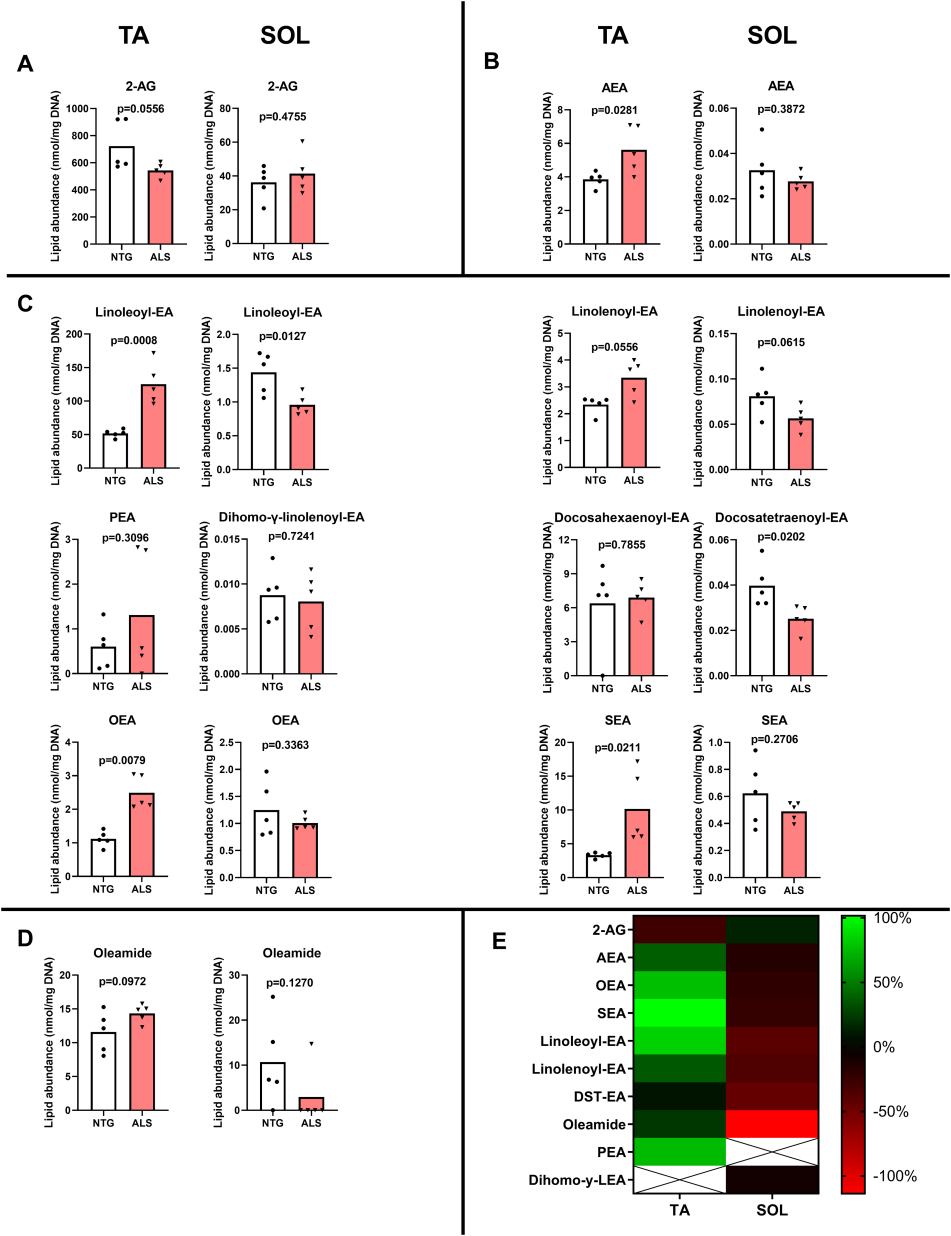
The effect of hSOD1 genotype on the endocannabinoid (congener) abundance in the tibialis anterior (TA) and soleus muscle (SOL). Endocannabinoid(s) (congeners) include 2‐arachidonoylglycerol (2‐AG; panel A), anandamide (AEA; panel B), the NAE congeners (panel C): palmitoylethanolamide (PEA), oleoylethanolamide (OEA), stearoylethanolamide (SEA), linoleoyl ethanolamide (linoleoyl‐EA), linolenoyl‐ethanolamide (linolenoyl‐EA), docosatetraenoyl‐ethanolamide (docosatetraenoyl‐EA or DST‐EA; panel H), dihomo‐γ‐linolenoyl‐ethanolamide (dihomo‐γ‐linolenoyl‐EA), and oleamide (panel D) and; panel H). The individual (dots and triangles) and average (bar) values of nontransgenic control mice (NTG; dots) and hSOD1‐transgene mice (ALS; triangles) are presented. *p* values of unpaired Student's *t*‐test (normally distributed) or Mann–Whitney U‐test (not normally distributed). The heat map (panel E) shows the %‐difference in endocannabinoid (congener) muscle abundance between the hSOD1‐transgene and nontransgenic control mice, for the TA and SOL (*n* = 5/group).

**FIGURE 5:**
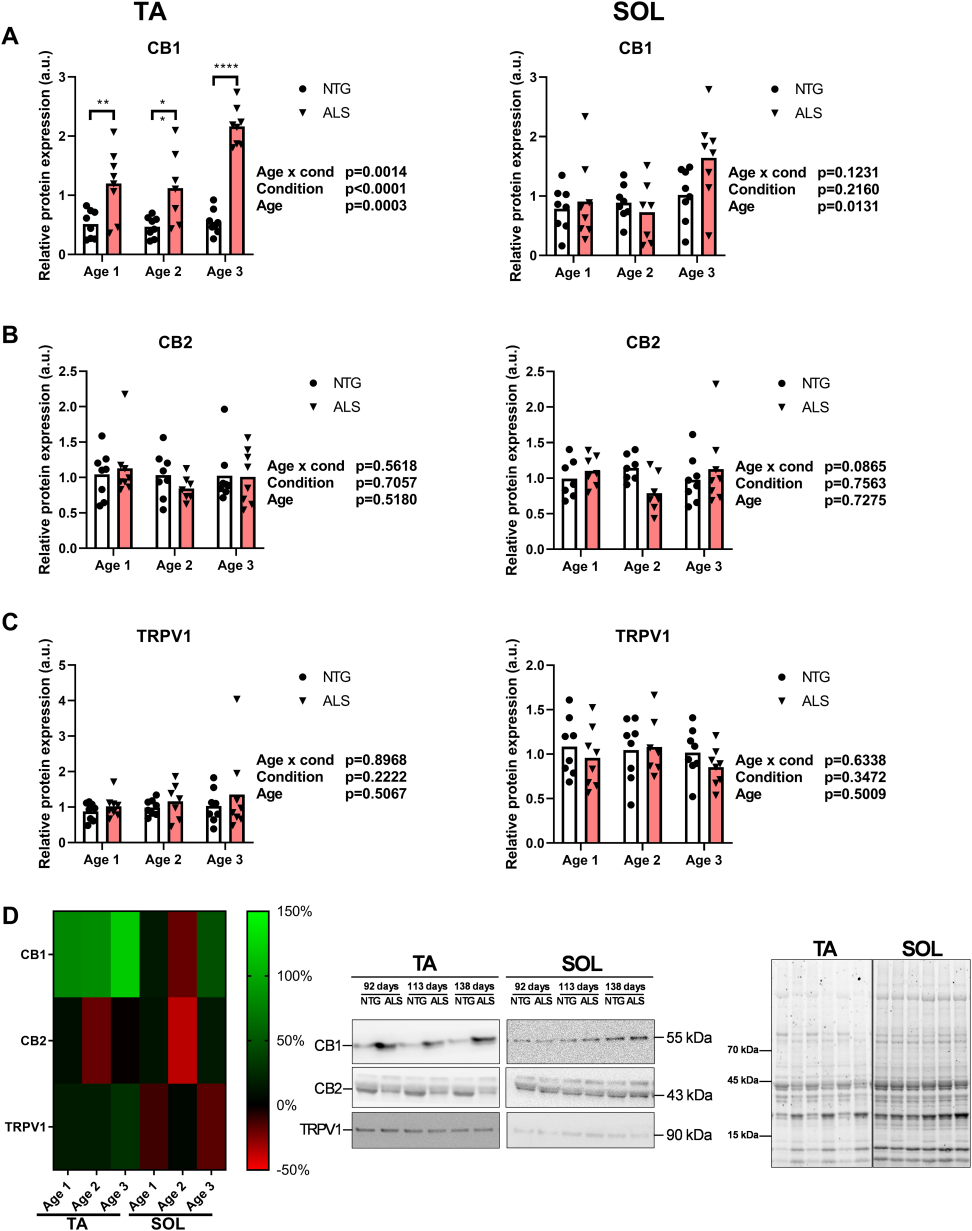
The effect of hSOD1 genotype and age on cannabinoid receptors in the tibialis anterior (TA) and soleus muscle (SOL). Cannabinoid receptors include cannabinoid receptor 1 (CB1; panel A), cannabinoid receptor 2 (CB2; panel B) and transient receptor potential vanilloid‐1 (TRPV1; panel C). The individual (dots and triangles) and average (bar) values of nontransgenic control mice (NTG; dots) and hSOD1‐transgene mice (ALS; triangles) are presented. *p* values of two‐way analysis of variance, and Bonferroni‐corrected post hoc analyses are presented. The heat map (panel D) shows the %‐difference in muscle cannabinoid receptor expression between the hSOD1‐transgene and nontransgenic control mice, for the TA and SOL and at three ages (age 1: 92 days, age 2: 113 days, age 3: 138 days) (*n* = 7–8/group).

**FIGURE 6:**
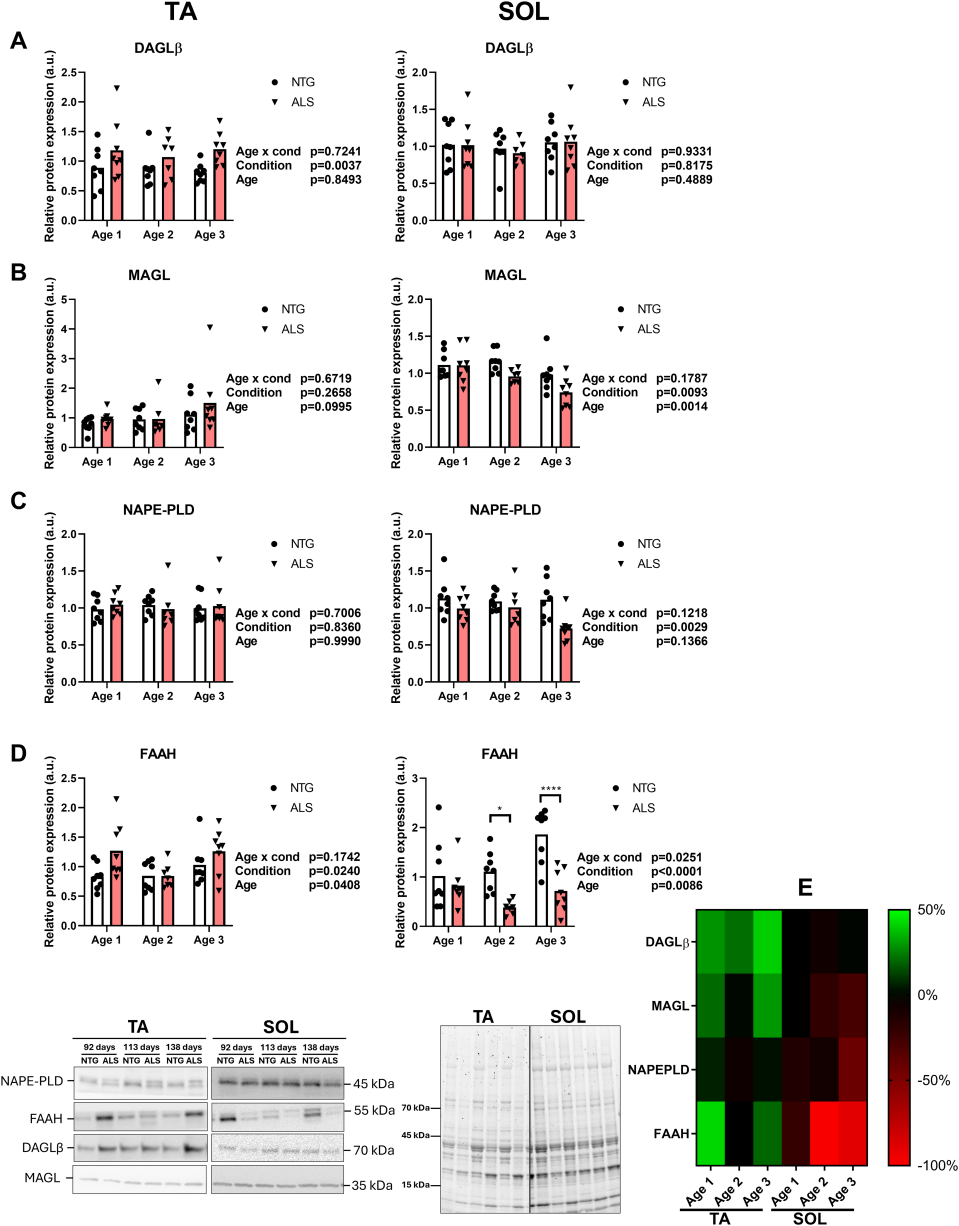
The effect of hSOD1 genotype and age on enzymes, responsible for endocannabinoid(−like mediator) synthesis and breakdown in the tibialis anterior (TA) and soleus muscle (SOL). Enzymes include diacylglycerol lipase β (DAGLβ; panel A), monoacylglycerol lipase (MAGL; panel B), *N*‐acyl phosphatidylethanolamine phospholipase D (NAPE‐PLD; panel C) and fatty‐acid amide hydrolase 1 (FAAH; panel D). The individual (dots and triangles) and average (bar) values of nontransgenic control mice (NTG; dots) and hSOD1‐transgene mice (ALS; triangles) are presented. *p* values of two‐way analysis of variance, and Bonferroni‐corrected post hoc analyses are presented. The heat map (panel E) shows the %‐difference in muscle enzyme expression between the hSOD1‐transgene and nontransgenic control mice, for the TA and SOL and at three ages (age 1: 92 days, age 2: 113 days, age 3: 138 days) (*n* = 7–8/group).

ALS also induced pronounced dysregulation of metabolic, stress and myogenic pathways in a muscle type–specific manner, with the TA showing extensive activation of catabolism (autophagy and ubiquitin‐proteasome system), anabolic signalling and inflammation, while the SOL displayed only modest and selective changes (Supporting Results; Figure 1, 2, 3, 4, 5, 6). Overall, molecular alterations closely paralleled the greater degree of muscle wasting observed in the TA.

### 3.2 Muscle Type–Specific Remodelling of (Inflammatory) Lipid Mediators in the Muscle of ALS Mice

Lipid remodelling is increasingly acknowledged as a central pathological hallmark of neuropathies [2], such as ALS [4, 29, 30], but the abundance and role of bioactive lipid mediators have never been determined in ALS skeletal muscle. Therefore, a targeted lipidomic approach revealed the lipid mediator abundance in the TA and SOL of ALS and NTG mice. Principal component analyses showed distinct lipid remodelling in ALS and NTG mice, in both muscles (Supporting Information). Unsaturated fatty acids serve as important precursors of inflammatory lipid mediators via enzymatic conversion (e.g., cyclooxygenase or COX, lipoxygenase or LOX, CYP450‐epoxygenase or CYP). In the TA, unsaturated fatty acids were generally more abundant in ALS versus NTG, whereas, in SOL, they were lower or unaffected by ALS (Figure 3A). Downstream lipid mediator species, including hydroxy fatty acids (HFAs) and epoxy fatty acids (EFAs), exhibited a very similar pattern, that is, higher abundance in the TA of ALS mice versus NTGs, and a lower abundance or no difference in the SOL of ALS mice (Figure 3B,C). HFAs and EFAs included arachidonic metabolites (HFA: HETEs, HHTs; EFA: EpETrE), eicosapentaenoic acid (EPA) metabolites (HFA: HEPEs; EFA: EpETEs), docosahexaenoic acid (DHA) metabolites (HFA: HDoHEs; EFA: EpDPE), linoleic acid metabolites (HFA: diHOMEs; EFA: EpOME) and α‐linolenic acid metabolites (HFA: HOTrEs). The abundance of prostaglandins, leukotrienes and oxo fatty acids was less consistently affected by ALS and did not differ between both muscle types (Figure 3D,E). Overall, ALS had a larger effect on the TA muscle lipidome than on the SOL lipidome, as 62.5% (TA) and 22.2% (SOL) of the (inflammatory) lipid mediators were significantly different in ALS compared to NTG. In addition, lipid remodelling was opposite in both muscles.

### 3.3 Muscle‐Specific and Temporal Endocannabinoid Remodelling in ALS

Endocannabinoids—Similar to the EFAs and HFAs, endocannabinoids (eCBs) and *N*‐acyl‐ethanolamine congeners were more affected in the TA of ALS versus NTG, than in the SOL. More precisely, in TA of ALS mice, four eCB(s) (congeners) out of nine were significantly different and three tended to be different compared to NTG mice. In contrast, only three of nine eCB(s) (congeners) were affected by ALS in the SOL (Figure 4). In the TA, different eCB (congeners) that share a common synthetic (e.g., via NAPE‐PLD) and degradation pathway (e.g., via FAAH) showed (a tendency towards) increased abundance in the ALS versus NTG muscle, that is, AEA (+37.3%; Figure 4B), OEA (75.9%; Figure 4C), SEA (+101.5%; Figure 4C), linoleoyl‐EA (LEA) (+82.9%; Figure 4C), linolenoyl‐EA (+35.2%; Figure 4C) and oleamide (+21.4%; Figure 4D). In contrast, 2‐AG, that does not share major metabolic pathways with AEA and the congeners, tended to be less abundant in ALS TA (−28.3%; Figure 4A). In the SOL, there was (a tendency towards) a lower abundance of LEA (−40.1%; Figure 4C), linolenoyl‐EA (−35.5%; Figure 4C) and docosatetraenoyl‐EA (−44.8%; Figure 4C) in ALS compared to NTG. Overall, the TA of ALS mice exhibited a severe endocannabinoid remodelling pattern, which was opposite to the moderate remodelling in the SOL (Figure 4E).

Receptors—CB1, but not CB2 and TRPV1, were affected by ALS. CB1 was significantly higher in the TA of ALS mice compared to NTG (+93%) and also tended to be higher with advancing age in ALS, whereas it remained stable in NTGs (*p*_interaction_ = 0.0014) (Figure 5A). None of the receptors were different between ALS and NTG in SOL.

Enzymes—DAGLβ expression, responsible for 2‐AG synthesis, was higher in ALS compared to NTG in the TA (+30.1%) but not in SOL (Figure 6A). MAGL expression, responsible for 2‐AG degradation, was lower in SOL (−15.9%), but not TA, of ALS mice compared to NTG mice (Figure 6B). NAPE‐PLD, which is responsible for the synthesis of AEA and *N*‐acyl‐ethanolamine congeners, remained unaffected in the TA by ALS, whereas it was lower expressed in the SOL of ALS versus NTG mice (−20.9%; Figure 6C). The AEA and *N*‐acyl‐ethanolamine congener degrading enzyme FAAH was higher expressed in the TA of ALS compared to NTG mice (+20.5%), whereas its expression was lower in the SOL of ALS mice (−69.1%; Figure 6D).

Surprisingly, endocannabinoid enzyme expression in the TA of ALS mice does not align with endocannabinoid abundance, that is, higher AEA and congener abundance versus unaffected NAPE‐PLD (synthesis) and higher FAAH (degradation) expression, and lower 2‐AG abundance versus higher DAGLβ (synthesis) expression and unaffected MAGL expression (degradation), which might be explained by presymptomatic muscle remodelling in ALS (*infra*).

Interestingly, transcriptomic analyses in skeletal muscle of ALS patients revealed that the same endocannabinoid targets were affected as in the TA of the murine ALS model, be it in an opposite direction. FAAH (~twofold; *p*_FDR_ = 0.010) as well as DAGL (~1.7‐fold; *p*_FDR_ < 0.0001) expression was lower in skeletal muscle of ALS patients compared to healthy controls (Table 1). This indicates that endocannabinoid remodelling in skeletal muscle is a well‐preserved adaptation in ALS, as well as in other muscle degenerative conditions [15, 16, 17, 18, 31].

**TABLE 1:** Endocannabinoid system‐related readouts of RNA sequencing in skeletal muscle tissue of human ALS patients versus age‐matched healthy controls.

Function	Gene	Gene ID	Log2FC	*p*	*p*_FDR_
AEA synthesis	NAPEPLD	222 236	−0.045	0.644	0.797
**AEA breakdown**	**FAAH**	**2166**	**−0.950**	**0.001**	**0.010**
AEA breakdown	FAAH2	158 584	+0.216	0.617	0.414
AEA breakdown	NAAA	27 163	−0.118	0.766	0.599
**2‐AG synthesis**	**DAGLA**	**747**	**−0.810**	**1.62 E** ^**−5**^	**< 0.0001**
**2‐AG synthesis**	**DAGLB**	**221 955**	**−0.636**	**6.53 E** ^**−7**^	**< 0.00001**
2‐AG breakdown	MGLL	11 343	−0.183	0.250	0.449
Cannabinoid receptor	CNR1	1268	−0.571	0.054	0.159
Cannabinoid receptor	CNR2	1269	+0.173	0.794	0.893
Cannabinoid receptor	TRPV1	7442	+0.325	0.009	0.043

Abbreviations: 2‐AG, 2‐arachidonoyl glycerol; AEA, anandamide.

### 3.4 Presymptomatic (Endocannabinoid) Remodelling in the Tibialis Anterior of ALS Mice

Because the mass of most muscles and molecular regulators was already affected at age 1 (92 days), muscle weight and molecular regulators were assessed in the TA of ALS and NTG mice at presymptomatic age (56 days). Also at this presymptomatic age, ALS mice already exhibited lower TA (−47.8%; *p* < 0.0001), SOL (−17.4%; *p* = 0.0036) and GAS (−44.8%; *p* < 0.0001), but not EDL muscle weight compared to NTG (Figure S7). Interestingly, ALS affected markers of muscle catabolism (increase in LC3b‐I, LC3b‐II and p62), muscle anabolism (increase in p‐S6rp), muscle stress (increase in caspase‐3) and myogenicity (increase myogenin and decrease in eMyHC) already at this young age (Figure S8). These findings confirm that muscle degenerative processes already occur before neurological symptoms, suggesting a reciprocal role for skeletal muscle in its communication towards motor neurons. Therefore, skeletal muscle tissue might be an interesting (early and adjuvant) therapeutic target to extend health span and improve quality of life in ALS.

To understand whether skeletal muscle endocannabinoid remodelling also precedes neurological symptoms, the protein expression of CB1 and endocannabinoid enzymes was also analysed at a presymptomatic age in the TA muscle of NTG and ALS mice. In line with findings at symptomatic age, CB1 was significantly higher expressed in presymptomatic ALS mice versus NTG (+75.8%; *p* = 0.0104; Figure S9A). DAGLβ expression in the TA of ALS mice was significantly upregulated versus NTGs at symptomatic ages, but was not affected at the presymptomatic age (Figure S9B). Interestingly, FAAH expression, which was significantly higher expressed at symptomatic ages, was significantly lower expressed in the TA of presymptomatic ALS mice (−68.2%; *p* = 0.0408; Figure S9E).

### 3.5 Peripherally Restricted Pharmacological FAAH Inhibition Does Not Improve Survival or Functional Capacity in ALS Mice

**FIGURE 7:**
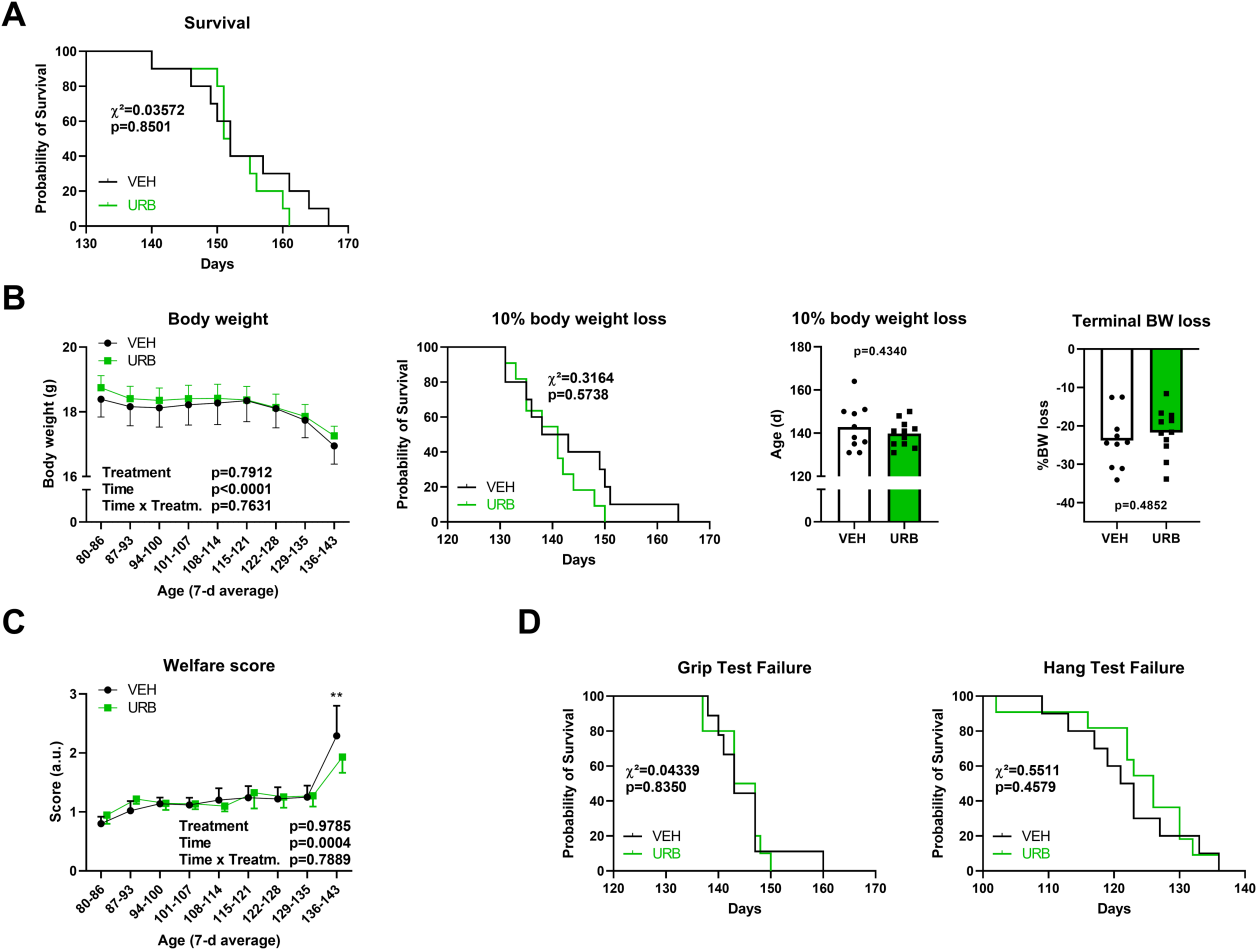
The effect of FAAH inhibition via URB937 treatment (1 mg/kg body weight intraperitoneally; 5×/week; *n* = 11), compared to vehicle treatment (VEH; *n* = 10), on survival (panel A), body weight (loss) (panel B), welfare score (panel C) and functional capacity (panel D) in female hSOD1‐transgene mice. Survival curves were analysed via the Gehan‐Breslow‐Wilcoxon test, body weight loss was assessed via Student's *t*‐test after normality testing, and longitudinal group comparisons were analysed via two‐way repeated measures analysis of variance. Time effects compared to baseline (80–86 days) are indicated by *p < 0.05, **p < 0.01, ***p < 0.001, ****p < 0.0001 in panels B and H (*n* = 10–11/group except for *n* = 9 of grip strength failure of VEH mice).

The muscle‐specific FAAH expression pattern (i.e., increased in ALS TA and decreased in ALS SOL at symptomatic age) and the temporal FAAH expression pattern (i.e., −68% expression in ALS TA at presymptomatic age and +42% in ALS at age 1) suggest that FAAH might play an important role in ALS‐related skeletal muscle atrophy, which is also confirmed with transcriptomic data of human skeletal biopsies of ALS patients [28]. Indeed, murine skeletal muscle FAAH expression was also increased during injury (+166%) [17], age‐related sarcopenia (+138%) [15] and cancer cachexia (+160%) [18]. Interestingly, genetic FAAH ablation improved motor neuron and motor unit survival as well as tetanic force [25]. Altogether, these findings indicate that FAAH is a promising pharmacological target to improve skeletal muscle health. This is particularly relevant in a murine hSOD1^G93A^ transgenic ALS model that suffers from early muscle atrophy, prior to the development of neurological symptoms. Therefore, we targeted FAAH levels with the peripherally restricted FAAH inhibitor URB937 (1 mg/kg/d) [23]. Compared with vehicle‐treated ALS mice, URB937 treatment did not affect any measures related to survival (Figure 7A), body weight (loss) (Figure 7B), welfare score (Figure 7C), functional motor capacity (Figure 7D), including grid hanging and grip strength, muscle mass (Figure S10), or molecular signalling in skeletal muscle (Figure S11).

## 4 Discussion

The present study provides the first comprehensive characterization of skeletal muscle lipid mediators, including ECS, remodelling in the hSOD1^G93A^ mouse model of ALS, which is paralleled by protein expression profiles. Transcriptomic data in skeletal muscle samples of patients revealed that the same ECS enzymes, that is, DAGL and FAAH, were also affected by ALS, but in an opposite direction [28]. ALS induces profound, muscle type–specific and disease stage‐dependent alterations in endocannabinoid and inflammatory lipid mediator abundance and in key regulators of muscle metabolism, myogenesis and cellular stress. Importantly, our work shows that ECS remodelling is not merely a consequence of advanced neuromuscular degeneration, but is already present at presymptomatic ages, supporting a potential role for skeletal muscle in the early pathophysiology of ALS.

### 4.1 ALS Induces Differential Vulnerability and Molecular Remodelling in Fast Versus Slow Muscles

Consistent with classic descriptions of the hSOD1^G93A^ model, the severe atrophy occurs in predominantly fast‐twitch muscles [32] such as the TA, while the predominantly slow‐twitch SOL was relatively preserved. This divergent vulnerability was accompanied by marked differences in protein metabolism, myogenic regulation, oxidative and apoptotic stress and lipid mediator abundance (e.g., 78% of the endocannabinoid (congeners) were/tended to be lower in the TA of ALS compared to NTG, whereas this was only 33% in the SOL).

The TA displayed a maladaptive co‐activation of catabolic and anabolic signalling pathways at both early and late stages of disease. Increased expression of autophagy markers (i.e., LC3b‐II/I, p62 and ATG5–12) together with heightened mTOR–S6rp–4E‐BP1 signalling suggests an unsuccessful compensatory attempt to preserve muscle mass despite ongoing wasting, a phenomenon also reported in other forms of muscle degeneration, such as cancer cachexia [18], sarcopenia [33], immobilization [15] and injury [17, 34]. The SOL displayed only modest alterations in these pathways. In addition, the myogenic signature was also affected by ALS [35], but diverged between muscles, that is, ALS TA showed reduced Myf5 (early; satellite cell activation) and eMyHC (late; myofibre maturation) but increased myogenin (late; differentiation) expression, while ALS SOL displayed suppression of Pax7 (early; stem cell marker) and a trend towards lower myogenin. ALS‐related myogenic remodelling (i.e., increased myogenin and decreased eMyHC expression) was already present in the presymptomatic TA, suggesting that this, together with a pro‐inflammatory environment (increased expression of the macrophage marker F4/80), resulted in an impaired regenerative environment, which potentially contributes to the early onset of muscle atrophy in ALS mice. Together, these findings highlight that ALS induces early, muscle type–specific disruptions of muscle quality control systems, which may partially explain the differential atrophy, in addition to neurological factors.

### 4.2 ALS Drives Early and Extensive Remodelling of Lipid Mediators and Endocannabinoid Tone in Skeletal Muscle

ALS profoundly disrupted lipid mediator networks in a muscle type–specific manner. The TA exhibited broad elevations in unsaturated fatty acids and downstream hydroxy and epoxy fatty acid derivatives, consistent with an inflammatory and oxidative lipid environment. In contrast, the SOL tended to show reduced or unchanged levels of these lipid mediators. This suggests a heightened inflammatory and metabolic stress burden in fast‐twitch muscles, fitting with their greater functional decline.

Interestingly, classical endocannabinoids and their *N*‐acyl‐ethanolamine congeners were also significantly remodelled. In the TA, multiple *N*‐acyl‐ethanolamines (AEA, OEA, SEA, LEA, linolenoyl‐EA and oleamide) were markedly elevated, whereas 2‐AG tended to be reduced. This pattern resembles ECS dysregulation reported in other degenerative muscle conditions such as Duchenne muscular dystrophy [16], injury [17] and cancer cachexia [18], suggesting a conserved response to muscle degeneration. The largely opposite remodelling observed in the SOL reinforces the idea that ECS changes reflect the degree of local muscle stress and vulnerability.

Surprisingly, endocannabinoid enzyme expression did not align in a straightforward manner with endocannabinoid (congener) abundance. FAAH protein levels were downregulated presymptomatically in TA but upregulated at symptomatic age, while NAPE‐PLD remained unchanged. These data support a dynamic regulation of ECS metabolism during disease progression rather than a static defect. The finding that FAAH is elevated in degenerating TA muscle at symptomatic age aligns with prior reports of increased FAAH in preclinical muscle degeneration models [15, 17, 18, 31]. The upregulation of CB1, particularly at presymptomatic age, further underscores the possibility that the ECS contributes to early pathophysiological signalling in ALS muscle, potentially through regulation of myogenicity [16, 17], (protein) metabolism [19, 20], apoptosis [17] and inflammatory [15] tone.

Overall, these observations support the concept that skeletal muscle participates in early nonneural pathology in ALS, echoing growing evidence that muscle mitochondrial dysfunction [36], metabolic deficits [37] and impaired regeneration [38] precede motor neuron degradation [39]. Such early alterations may contribute to a feed‐forward cycle in which a degenerative muscle environment contributes to neuromuscular junction instability [40] and enhances motor neuron stress [41].

### 4.3 Pharmacological FAAH Inhibition Does Not Improve Survival or Functional Endpoints in ALS Mice

Given the temporal and muscle‐specific FAAH expression profile, the effect of peripheral FAAH inhibition on disease progression was evaluated in ALS mice. In the present study, chronic URB937 administration from the age of 80 days did not improve survival, body weight maintenance or functional performance. The peripheral FAAH inhibition monotherapy might be insufficiently strong to fully reverse the degenerative muscle profile, characterized by altered protein metabolism, myogenicity, inflammation and apoptosis, despite the fact that all these systems have been related to the ECS [16, 17, 19, 42].

In contrast, earlier evidence showed that genetic or pharmacological FAAH inactivation could improve muscle functionality in ALS mice [25, 43]. Treatment with the FAAH inhibitor PF‐04457845 from the age of 56 days increased survival and prevented weight loss and functional decline in hSOD1^G93A^ mice [43]. This could be, at least partially, explained by improved preservation of motor neurons within the lumbar spinal cord [43]. In addition, genetic FAAH ablation preserved muscle strength, motor neuron number and motor unit number, but did not affect survival in ALS mice [25]. Therefore, it can be hypothesized that approaches that target systemic FAAH (including central tissues) might hold a stronger therapeutic profile when compared to peripherally restricted FAAH inhibition, as was done in the present study. AEA levels were indeed elevated in the spinal cord of ALS mice at symptomatic ages (90 and 120 days) but not presymptomatic ages (40 days) [25]. In line with the neuroprotective effects of endocannabinoids [44, 45, 46], the authors suggested that increased AEA levels could be a protective response to counteract disease progression [25]. Systemic FAAH inhibition, via PF‐04457845 treatment, further increased AEA and other *N*‐acyl‐ethanolamine congeners in the spinal cord of ALS mice [43]. This increase, together with improved survival and motor function, indicates that ECS remodelling in the spinal cord remains critical for disease progression in ALS mice. Future studies should therefore explore the role of the ECS and of FAAH in central tissues in neurodegenerative conditions, whereas FAAH modulation might remain an important target in muscle‐centred degenerative conditions, such as cancer cachexia [18], sarcopenia [15] or injury [17].

### 4.4 Considerations and Limitations

To allow proper interpretation of the obtained study results, some considerations should be taken into account.

First, the hSOD1^G93A^ transgenic mouse line is a well‐established model that replicates different features of the ALS pathogenesis observed in humans, such as progressive lower motor neuron degeneration, neuroinflammation and motor weakness [47, 48]. However, the severe transgene expression results in artificially accelerated disease progression and pathological features that are not typical in human ALS [49]. This difference divergent disease and muscle degeneration progression might also have contributed to opposite changes in FAAH and DAGL expression between human patients with ALS and the murine model. Regarding the pathophysiology in ALS‐related muscle atrophy, there are indications that there are considerable parallels between the hSOD1^G93A^ model and human ALS muscle [28]. Most evidence in human ALS muscle points towards alterations in myogenic engagement and myofibre growth [50] and towards metabolic deficits [50], for example, mitochondrial capacity, which is similar to the present and earlier findings [51]. However, there is definitely a need for more longitudinal (including early stage) evidence as well as muscle type–specific evidence (primarily fast‐ vs. slow‐twitch muscles) in humans to confirm parallels with the present study (e.g., lipid mediator remodelling and apoptosis) and to eventually gain better insights into the pathophysiology underlying ALS‐related muscle atrophy.

Second, despite similarities in the pathophysiology of muscle atrophy between different forms of familial ALS (e.g., proteostasis stress and mitochondrial dysfunction), it may be that the hSOD1^G93A^‐related muscle atrophy in the present study does not fully parallel atrophy of other ALS forms or occurs at a different progression rate. For instance, whereas SOD1^G93A^ ALS muscle atrophy is driven mainly by metabolic and mitochondrial dysfunction (e.g., swelling), hypermetabolism and early NMJ denervation, the degeneration process in ALS due to FUS mutations is driven by early toxic mislocalization of FUS, eventually resulting in decreased lipid metabolism and direct mitochondrial damage, producing a strong muscle‐intrinsic degenerative phenotype [52].

Third, only female mice were included in this work since they exhibit a delayed disease onset and longer lifespan in the hSOD1G93A model, resulting in a less compressed disease course that facilitates the detection of age‐dependent molecular changes in skeletal muscle. In contrast, male SOD1^G93A^ mice often show earlier onset and faster progression, further accentuating the already accelerated phenotype of this model relative to human ALS, which is typically a late‐onset disease with a more gradual progression (median survival of 2–5 years from symptom onset) [53, 54]. While this makes females somewhat more suitable for capturing temporal disease dynamics, it should be noted that ALS is more frequently diagnosed in men, indicating a complementary limitation regarding disease incidence. Furthermore, biological sex is directly relevant for ALS progression, skeletal muscle physiology and endocannabinoid tone. Sex hormones such as oestrogens and androgens such as testosterone can modulate ALS risk and progression by affecting motor neuron survival, neuroinflammation and synaptic plasticity, with oestrogens showing neuroprotective effects in preclinical models, and androgens showing more context‐specific actions depending on their concentration and timing [55, 56]. In addition to neural effects, sex (hormones) can also modulate muscle molecular signalling at the level of metabolism, myogenicity and inflammation [57]. Finally, the ECS itself is sexually dimorphic, as cannabinoid receptor expression, endocannabinoid levels and their metabolic regulation are influenced by sex hormones [58]. Therefore, results from the present study should be treated with caution when extrapolating to the broader patient population, and future studies directly comparing both sexes are warranted.

Fourth, the URB937 treatment (study 2) started at the age of 80 days, which corresponds with the onset of the first motor deficits. Since muscle atrophy was already present at presymptomatic age (56 days), the treatment might have been more impactful when started at a younger age. Indeed, early‐onset treatment with the FAAH inhibitor PF‐04457845 (56 days) improved survival and motor functional decline [43]. However, presymptomatic treatment is not recommended from a translational point of view, as preclinical success at presymptomatic ages does not necessarily result in clinical success [59].

Fifth, lipid mediator analyses were performed at one age and in one tissue. Given the temporal dynamics and the whole‐body character of the ALS pathophysiology, future work should examine longitudinal and multitissue lipid mediator remodelling (e.g., nervous system, blood) to gain a better understanding of the complex underlying mechanisms. Inflammatory cytokines and inflammatory monocytes are increased in the circulation of hSOD1^93A^ mice, even at presymptomatic age (40–50 days) [60, 61]. This systemic inflammation might drive early muscle inflammation and inflammatory lipid remodelling, for example, through cyclooxygenase‐2, which is induced by inflammatory stimuli. In addition, the lipidomic pipeline only included low‐abundant bioactive lipid mediators and not high‐abundant lipids such as phospholipids and sphingolipids. It should be noted that these lipid classes are also affected in muscle degenerative conditions, such as neuromuscular disease [5], unloading [62], aging [63], metabolic disease[64], muscular dystrophy[65], where they serve as structural components and regulators of metabolic control (e.g., insulin resistance), satellite cell growth and contractile function (e.g., via calcium handling). Future studies that integrate temporal lipid profiling of low‐ and high‐abundant lipid classes and neuromuscular status might provide new insights in how the muscle lipidome associates with functional symptoms (e.g., muscle strength and neuromuscular communication) and molecular signalling in neuromuscular disease.

## 5 Conclusions

This study made use of a between‐condition (ALS vs. healthy controls) and between‐muscle type (tibialis anterior vs. soleus) comparison to identify robust, muscle‐specific and temporal ECS remodelling as a previously overlooked component of ALS skeletal muscle pathology. Fast‐twitch muscle exhibits extensive and early dysregulation of inflammatory (lipid) mediators, endocannabinoids and their biosynthetic/degradative enzymes. Although pharmacological, peripherally restricted FAAH inhibition alone was not sufficient to improve lifespan and motor symptoms in female hSOD1^G93A^ mice, our findings suggest that the ECS represents an integral part of the degenerative landscape of the ALS muscle. Future studies should explore whether therapeutic interventions that target the lipid mediator networks, metabolic pathways or endocannabinoid signalling can synergistically support muscle health and neuromuscular stability. Ultimately, understanding the reciprocal communication between early muscle atrophy and vulnerable motor neurons may open new avenues for early and adjunctive ALS therapeutics.

## Funding

The study was supported by the Research Foundation Flanders (FWO; G086823N). Sebastiaan Dalle has received postdoctoral fellowships (12Z8622N from Research Foundation Flanders [FWO] and PDMt1/24/001 from KU Leuven Research Council) and Moniek Schouten has received a PhD fellowship (11PRA24N from Research Foundation Flanders [FWO]).

## Ethics Statement

These mouse studies were approved by KU Leuven Animal Ethics Committee (M022/2023 and BW‐034/2025). The study from which human data we reused was approved by the University of Alabama at Birmingham (UAB) Institutional Review Board (IRB‐100908007 and IRB‐091222037). All persons gave their informed consent prior to their inclusion in the study.

## Conflicts of Interest

L.V.D.B. is head of the Scientific Advisory Board of Augustine Therapeutics (Leuven, Belgium) and is part of the Investment Advisory Board of Droia Ventures (Meise, Belgium).

## Supporting information


**Table S1:** Functional characteristics of selected ages in ALS mice.
**Figure S1:** The effect of hSOD1 genotype and age on autophagy markers in the tibialis anterior (TA) and soleus muscle (SOL). Autophagy markers include microtubule‐associated proteins 1A/1B light chain 3B (cytosolic LC3b‐I and autophagosomal LC3b‐II; panel A–C), p62 (panel D) and autophagy related 5–12 (ATG5–12; panel E). The individual (dots and triangles) and average (bar) values of nontransgenic control mice (NTG; dots) and hSOD1‐transgene mice (ALS; triangles) are presented. Age 1, 2 and 3 refer to an age of 92, 113 and 138 days, respectively. *p* values of two‐way analysis of variance, and Bonferroni‐corrected post hoc analyses are presented (*n* = 7–8/group).
**Figure S2:** The effect of hSOD1 genotype and age on the ubiquitin proteasome markers muscle RING‐finger protein‐1 (MuRF1) and atrogin 1 in the tibialis anterior (TA) and soleus muscle (SOL). The individual (dots and triangles) and average (bar) values of nontransgenic control mice (NTG; dots) and hSOD1‐transgene mice (ALS; triangles) are presented. Age 1, 2 and 3 refer to an age of 92, 113 and 138 days, respectively. *p* values of two‐way analysis of variance (*n* = 7–8/group).
**Figure S3:** The effect of hSOD1 genotype and age on anabolic markers in the tibialis anterior (TA) and soleus muscle (SOL). Anabolic markers include phosphorylated mammalian target of rapamycin (p‐mTOR; panel A), phosphorylated S6 ribosomal protein (p‐S6rp; panel B), phosphorylated Eukaryotic translation initiation factor 4E‐binding protein 1 (p‐4E‐BP1; panel C) and phosphorylated eukaryotic translation elongation factor 2 (p‐eEF2; panel D). The individual (dots and triangles) and average (bar) values of nontransgenic control mice (NTG; dots) and hSOD1‐transgene mice (ALS; triangles) are presented. Age 1, 2 and 3 refer to an age of 92, 113 and 138 days, respectively. *p* values of two‐way analysis of variance, and Bonferroni‐corrected post hoc analyses are presented (*n* = 7–8/group).
**Figure S4:** The effect of hSOD1 genotype and age on inflammatory and stress markers in the tibialis anterior (TA) and soleus muscle (SOL). Inflammatory and stress markers include the macrophage marker F4/80 (panel A), the apoptotic markers caspase‐3 (panel B) and Bax (panel C) and the endoplasmatic reticulum marker binding immunoglobulin protein (BiP; panel D). The individual (dots and triangles) and average (bar) values of nontransgenic control mice (NTG; dots) and hSOD1‐transgene mice (ALS; triangles) are presented. Age 1, 2 and 3 refer to an age of 92,113 and 138 days, respectively. *p* values of two‐way analysis of variance, and Bonferroni‐corrected post hoc analyses are presented (*n* = 7–8/group except for *n* = 6 for BiP of SOL in ALS age 2 and 3).
**Figure S5:** The effect of hSOD1 genotype and age on metabolic markers in the tibialis anterior (TA) and soleus muscle (SOL). Metabolic markers include phosphorylated acetyl‐CoA carboxylase (p‐ACC; panel A), fatty acid synthase (panel B) and peroxisome proliferator‐activated receptor‐gamma coactivator 1α (PGC1α; panel C). The individual (dots and triangles) and average (bar) values of nontransgenic control mice (NTG; dots) and hSOD1‐transgene mice (ALS; triangles) are presented. Age 1, 2 and 3 refer to an age of 92, 113 and 138 days, respectively. *p* values of two‐way analysis of variance, and Bonferroni‐corrected post hoc analyses are presented (*n* = 7–8/group except for *n* = 6 for p‐ACC in ALS age 2).
**Figure S6:** The effect of hSOD1 genotype and age on myogenic markers in the tibialis anterior (TA) and soleus muscle (SOL). Myogenic markers include paired box 7 (Pax7; panel A), myogenic factor 5 (Myf5; panel B), myogenin (panel C) and embryonal myosin heavy chain (eMyHC; panel D). The individual (dots and triangles) and average (bar) values of nontransgenic control mice (NTG; dots) and hSOD1‐transgene mice (ALS; triangles) are presented. Age 1, 2 and 3 refer to an age of 92, 113 and 138 days, respectively. *p* values of two‐way analysis of variance, and Bonferroni‐corrected post hoc analyses are presented (*n* = 7–8/group).
**Figure S7:** The effect of hSOD1 genotype at the presymptomatic age of 56 days on the absolute weight of the tibialis anterior muscle (panel A), the extensor digitorum longus muscle (extensor DL; panel B), the soleus muscle (panel C) and the gastrocnemius muscle (panel D). The individual (dots and triangles) and average (bar) values of nontransgenic control mice (NTG; dots) and hSOD1‐transgene mice (ALS; triangles) are presented. *p* values of unpaired *t*‐test (normally distributed) or Mann–Whitney *U* test (nonnormally distributed between both genotypes are presented (*n* = 8/group).
**Figure S8:** The effect of hSOD1 genotype at the presymptomatic age of 56 days on the protein expression of (p‐mTOR; panel A), (p‐S6rp; panel B), (p‐4E‐BP1; panel C), (p62; panel D), (LC3b‐I; panel E), (LC3b‐II; panel F), caspase‐3 (panel G), myogenin (panel H) and embryonal myosin heavy chain (eMyHC; panel I) in the tibialis anterior muscle compared to nontransgenic controls (NTG). The individual (dots and triangles) and average (bar) values of nontransgenic control mice (NTG; dots) and hSOD1‐transgene mice (ALS; triangles) are presented. *p* values of unpaired *t*‐test (normally distributed) or Mann–Whitney *U* test (nonnormally distributed) between both genotypes are presented (*n* = 8/group).
**Figure S9:** The effect of hSOD1 genotype at the presymptomatic age of 56 days on the expression of cannabinoid receptor 1 (CB1; panel A), diaglycerol lipase β (DAGLβ; panel B), monoacylglycerol lipase (MAGL; panel C), *N*‐acyl phosphatidylethanolamine phospholipase D (NAPE‐PLD; panel D) and fatty‐acid amide hydrolase 1 (FAAH; panel E) in the tibialis anterior muscle compared to nontransgenic controls (NTG). The individual (dots and triangles) and average (bar) values of NTG (dots) and hSOD1‐transgene mice (ALS; triangles) are presented. *p* values of unpaired *t*‐test (normally distributed) or Mann–Whitney *U* test (nonnormally distributed) between both genotypes are presented (*n* = 8/group).
**Figure S10:** The effect of FAAH inhibition via URB937 treatment (1 mg/kg body weight intraperitoneally; 5×/week; *n* = 9–10), compared to vehicle treatment (VEH; *n* = 6), on the absolute weight of the tibialis anterior muscle (TA), the gastrocnemius muscle (GAS) and the soleus muscle (SOL). Difference in muscle mass was assessed via Student's *t*‐test. The individual (dots and rectangles) and average (bar) values of nontransgenic control mice (NTG; healthy reference group of 135 days old) and of VEH‐treated and URB‐treated hSOD1‐transgene mice (ALS; triangles) are presented.
**Figure S11:** The effect of daily URB937 (U; 1 mg/kg/d) versus vehicle (V) treatment from the age of 80 days old until humane endpoints on the protein expression levels of markers of protein metabolism, muscle stress and neuromuscular junction in the tibialis anterior muscle of female hSOD193A. Tibialis anterior muscle samples from nontransgenic controls (C; 135 days old) were added as a healthy reference group. The vehicle‐ and URB937‐treated groups were compared via the Student's *t*‐test. The individual (dots, squares and triangles) and average (bar) values are presented. P‐mTOR, phosphorylated mechanistic target of rapamycin; CB1, cannabinoid receptor 1; LC3b, microtubule‐associated protein 1 light chain 3 beta; F4/80, Bax, Bcl‐2 associated X‐protein; MuSK, muscle‐specific kinase; NFH, neurofilament heavy chain; MuRF1, muscle‐specific RING finger protein 1; PGC1α, peroxisome proliferator‐activated receptor gamma coactivator 1‐alpha (*n* = 7–10/group).

## Data Availability

The data that support the findings of this study are available from the corresponding author upon reasonable request.
