# Supplementary material for: Early and Divergent Lipid Mediator Remodelling in Fast Versus Slow Skeletal Muscles of Female hSOD1G93A Mice
Source: J Cachexia Sarcopenia Muscle. 2026 Aug 4;17(4):e70357. doi: 10.1002/jcsm.70357 (PMC13437094; doi:10.1002/jcsm.70357)
Supplement: Supplementary file 1 [file JCSM-17-e70357-s001.docx]

**Early and Divergent Lipid Mediator Remodeling in Fast versus Slow Skeletal Muscles of Female hSOD1^G93A^ Mice**

Sebastiaan Dalle^1,2^*^,$^, Kaat Vanderbeke^1^*, Thibaut Burg^3,4^, Moniek Schouten^1^, Wout Lauriks^1^, Nicole Hersmus^3,4,5^, Ludo Van Den Bosch^3,4^, Katrien Koppo^1,$^

^1^Exercise and Muscle Physiology Research Group; Department of Movement Sciences; University of Leuven; Tervuursevest 101; 3001 Leuven; Belgium

^2^MOVANT Research Group; Department of Rehabilitation Sciences and Physiotherapy; University of Antwerp; Universiteitsplein 1; 2610 Wilrijk; Belgium

^3^Leuven Brain Institute; Department of Neurosciences; University of Leuven; ON5, Herestraat 49; 3000 Leuven; Belgium

^4^Laboratory of Neurobiology; VIB Center for Neuroscience; ON5, Herestraat 49; 3000 Leuven; Belgium

^5^Department of Neurology; University Hospitals Leuven; Herestraat 49; 3001 Leuven; Belgium

*contributed equally

^$^corresponding authors: [Sebastiaan.dalle@kuleuven.be](mailto:Sebastiaan.dalle@kuleuven.be); Sebastiaan.dalle@uantwerpen.be; Katrien.koppo@kuleuven.be

Supplementary data

Supplementary Table 1. Functional characteristics of selected ages in ALS mice.

|  | Age 1 (~92 days) | Age 2 (~113 days) | Age 3 (~138 days) |
| --- | --- | --- | --- |
| BW difference *vs.* NTGs | -8.7 % | -12.1 % | -28.1 % |
| Gastrocnemius muscle weight difference *vs.* NTGs | -67.0 % | -69.1 % | -93.3 % |
| Muscle strength loss *vs.* 80 days* | -3.4 % | -19.8 % | -54 % |
| Animal welfare score *vs.* 80 days* | 28.3 % | 31.0 % | 140 % |

BW: body weight; NTG: non-transgenic controls. *muscle strength & welfare score were assessed in the cohort of mice of study 2 (intervention study).

Protein extraction – Experiment 1

Smaller pieces from different parts of TA (15-20 mg in total; representing 30-50% of the entire muscle mass) and both SOLs (~13 mg) were homogenized 4 x 20 s in ice-cold lysis buffer (1:10 w/v; 50 mM Tris-HCl, pH 7.0; 270 mM sucrose; 5 mM ethylene glycol tetraacetic acid (EGTA); 1 mM ethylenediaminetetraacetic acid (EDTA); 1 mM sodium orthovanadate; 50 mM glycerophosphate; 5 mM sodium pyrophosphate; 50 mM sodium fluoride; 1 mM dithiothreitol; 0.1% Triton X-100; and a protease inhibitor tablet [cOmplete Mini EDTA-free; Merck, NJ, USA]) with 8 silicon beats using the FastPrep (MP Biomedicals, Santa Ana, CA, USA). Homogenates were centrifuged (10000 g for 25 min at 4 °C) and the supernatant was stored at -80 °C. Protein concentrations were assessed with the DC protein assay kit, applying a bovine serum albumin (BSA) protein standard (Bio-Rad Laboratories, Nazareth, Belgium). Lysis buffer was added to equalize the final protein concentrations, and laemmli (20% of the total volume) was added to obtain muscle lysates.

Western blot analysis – Experiment 1

Proteins (20-30 µg) were separated by SDS-PAGE on 4-20% Criterion TGX stain-free gels (Bio-Rad, Hercules, CA, USA) (50-60 min, 200 V) and transferred to polyvinylidene difluoride membranes (80 min, 400 mA). Hereafter, membranes were blocked in TBS-T (tris-buffered saline with Tween-20) containing 5% non-fat milk for 1h. Next, membranes were incubated overnight at 4 °C in TBS-T containing 5% non-fat milk or 5% BSA with primary antibodies that have been validated for specificity by the manufacturer and have been tested in-house. Membranes were then washed and incubated for 45 min at room temperature with the appropriate horseradish peroxidase-conjugated secondary antibody (1/7000; Sigma Aldrich, Bornem, Belgium). Upon incubation with SuperSignal Pico (#34577) or Femto (#34096) chemiluminescence substrate (Thermo Scientific, Waltham, MA, USA), membranes were scanned and quantified with Genesnap and Genetools Softwares (Syngene, Cambridge, UK). Results are presented as the protein of interest, relative to the total protein loading control. The total protein loading control was obtained via UV-light-induced tryptophan fluorescence on the TGX stain-free gel. The following primary antibodies were used:

- Endocannabinoid signaling: CB1 (ab259323; 1/400), CB2 (ab3561; 1/1000); transient receptor potential cation channel subfamily V member 1 (TRPV1; ab6166; 1/1000); N-acyl phosphatidylethanolamine-specific phospholipase D (NAPE-PLD; ab95397; 1/500); fatty-acid amide hydrolase 1 (FAAH; sc-100,739; 1/250); diacylglycerol lipase β (DAGLβ; CST-12574; 1/500); monoacylglycerol lipase (MAGL; ab24701; 1/200)
- Anabolism: p-mammalian target of rapamycin (p-mTOR^Ser2448^; CST–2971S; 1/1000), p-S6 ribosomal protein (p-S6rp^Ser235/236^; CST-2211; 1/1000), p-eukaryotic translation initiation factor 4E-binding protein 1 (p-4E-BP1^Thr37/46^; GTX-133182; 1/500), p-eukaryotic translation elongation factor 2 (p-eEF2^Thr56^; CST-2331; 1/1000)
- Myogenicity: paired box 7 (Pax7; DSHB; Iowa City, IA, USA; 1/100), myogenic factor 5 (Myf5; ab125301; 1/1000), myogenin (DSHB; 1/300), embryonal myosin heavy chain (eMyHC; DSHB; 1/1000)
- Metabolism: p-acetyl-CoA carboxylase (p-ACC^Ser79^; CST-3661; 1/1000), fatty acid synthase (FAS; sc-48357; 1/1000), peroxisome proliferator-activated receptor gamma coactivator 1-α (PGC1α; sc-517380; 1/500)
- Inflammation & stress: EGF-like module-containing mucin-like hormone receptor-like 1 (F4/80; CST-70076; 1/500), caspase-3 (CST-9662; 1/1000), bcl-2-associated X protein (Bax; GTX-635715; 1/500), binding immunoglobulin protein (BiP; CST-3177; 1/500)
- Autophagy: microtubule-associated proteins 1A/1B Light Chain 3B (LC3B; CST-3868; 1/1000), p62 (sequestosome 1; GP62-C; 1/1000), autophagy related protein 5-12 (ATG5-12; CST-4180S; 1/500)
- Structural elements: collagen (DSHB Col1a1 F1C3; 1/500), dystrophin (sc-365954; 1/1000)

Animal welfare scoring grid ALS mice

Appearance

0: smooth coat

1: patches of hair piloerected / ruffled fur

2: majority of the back is piloerected

3: piloerection may or may not be present, mouse appears "puffy"

4: piloerection may or may not be present, mouse appears emaciated

Activity

0: Normal amount of activity. Mouse is any of: eating, drinking, climbing, running, fighting

1: Slightly suppressed activity.

2: Suppressed activity. Mouse is stationary with occasional investigative movement

3: No activity. Mouse is stationary

4: No activity. Mouse experiencing tremors

Eyes

0: Open

1: Eyes not fully open, possibly with secretions

2: Eyes at least half closed, possibly with secretions

3: Eyes half closed or more, possibly with secretions

4: Eyes closed or milky

Pain & distress behavior

0: no vocalization when handled or touched, no self mutilation

2: slight vocalization when handled or touched, no self mutilation

4: excessive or abnormal vocalization when handled or touched, or self mutilation (biting or scratching)

Breathing

0: normal rhythm, rapid mouse respiration, with RR>120 (too fast to count)

1: RR>120, but with periods of laboured breating

2: slow respirations (RR:61-120)

4: persistent, difficult laboured breathing or continuous gasping or repiratory rate <60

Neurological scoring

0: Full extension of hind legs away from lateral midline when mouse is suspended by its tail

1: collapse or partial collapse of leg extension toward lateral midline (weakness) or trembling of hind legs during tail suspension

2: toes curl at least twice during walking of 30 cm, or any part of the foot is dragging along the cage bottom/table

3: Rigid paralysis or minimal joint movement. The foot is not being used for forward motion

4: Mouse cannot right itself up within 20 seconds after being placed on either side on a flat surface or severe parlysis

Weight loss over 7-day interval

0: <5%

1: 5-10%

2: 10-15%

3: 15-20%

4: >20%

RNA sequencing procedure and gene count data handling

RNA was isolated via TRIzol, and quality and quantity were assessed with Agilent RNA 6000 nano kit in 2100 Bioanalyzer System (Agilent)^1^. High-quality RNA (500 ng) was used to prepare the sequencing library (Illumina TruSeq Stranded Total RNA Library prep kit). After rRNA depletion and complementary deoxyribonucleic acid (cDNA) generation, cDNAs were subjected to 3’ end adenylation, adapter ligation and purification with AMPure beads (BeckmanCoulter). The products were size-selected with SPRIselect beads (Beckman Coulter), and the selected cDNAs were enriched by polymerase chain reaction (PCR) and repurified with SPRIselect beads to generate the final libraries. The quantity and quality of sequencing libraries were checked using Agilent DNA 1000 Screen Tape (Agilent TapeStation). Single-read sequencing was performed for 124 cycles with an Illumina NovaSeq 6000 sequencer. BCL files were de-multiplexed and converted to FASTQ files using bcl2fastq program (v2.20.0.422). FASTQ files were trimmed for adapter sequences using Cutadapt version v1.18 and aligned to human genome hg19 Ensembl v82 using STAR software v2.4.0j^2^, and featureCounts (v1.6.4)^3^ were used to create gene counts from the samples for RNA analysis.

1.	Tsitsipatis, D. *et al.* *Transcriptomic Analysis of Human ALS Skeletal Muscle Reveals a Disease-Specific Pattern of Dysregulated CircRNAs*. *AGING 2022* vol. 14 www.aging-us.comwww.aging-us.com (2022).

2.	Dobin, A. *et al.* STAR: Ultrafast universal RNA-seq aligner. *Bioinformatics* **29**, 15–21 (2013).

3.	Liao, Y., Smyth, G. K. & Shi, W. FeatureCounts: An efficient general purpose program for assigning sequence reads to genomic features. *Bioinformatics* **30**, 923–930 (2014).

Supplementary Results

Protein marker phenotyping reveals muscle-type-specific ALS-induced remodeling of muscle protein metabolism, stress, metabolism and myogenicity

We further confirmed muscle-type-specific degeneration in ALS on a molecular level by analyzing the protein expression levels of markers of metabolism, stress and myogenicity in the TA and SOL. Muscle mass is determined by the balance between muscle protein synthesis and muscle protein breakdown. Markers of autophagy, an important catabolic pathway, *i.e.* LC3b-I (+74%), LC3b-II (+76%), p62 (+61%) and ATG5-12 (+37%), were more highly expressed in the TA of ALS mice than in the TA of NTG mice (Suppl. Fig. 1A,B,D,E). In SOL, p62 expression was higher in ALS than in NTG (Suppl. Fig. 1D), whereas the expression of the other markers was not different between both conditions (Suppl. Fig. 1A,B,C,E). In addition to autophagic markers, also the ubiquitin-proteasome system marker MuRF1 was more highly expressed in the TA and SOL of ALS mice compared to NTG (Suppl. Fig. 2). The Akt-mTOR pathway is important for the activation of muscle protein synthesis. In the TA, p-mTOR (+75%; p<0.01; Suppl. Fig. 3A), p-S6rp (+58%; p<0.01; Suppl. Fig. 3B) and p-4E-BP1 (+52%; p<0.01; Suppl. Fig. 3C) were more expressed in ALS than in NTG, whereas p-eEF2 was lower expressed in ALS (-71%; p<0.01; Suppl. Fig. 3D). Interestingly, despite being already higher expressed in the ALS TA at age 1 (+55.6%) and 2 (+52.6%), the p-mTOR expression even further increased at age 3 *vs.* NTGs (+104.6%; p_interaction_=0.0054). In the SOL, p-S6rp (+46%) was more expressed in ALS than in NTG but none of the other anabolic markers were differently expressed between both conditions (Suppl. Fig. 3A,B,C,D). In line with previous evidence in muscle degenerative conditions^25,26^, pathway activation might be an attempt to compensate for muscle wasting without resulting in actual muscle protein synthesis.

Protein expression levels of some inflammatory and stress markers revealed higher expression of the macrophage marker F4/80 and the apoptotic markers caspase-3 and Bax in ALS compared to NTG in the TA and SOL (Suppl. Fig. 4A,B,C). For these markers, differences were more pronounced in the TA (+79% on average) than in the SOL (+41% on average; p=0.04). Unexpectedly, BiP expression, which typically responds to endoplasmic reticulum stress, was lower in ALS *vs.* NTG in the TA (-48%; p<0.01), and in the SOL, but only at the highest age (-45%; p<0.01) (Suppl. Fig. 4D).

In the TA, ALS resulted in higher expression of promoters of fatty acid metabolism, *i.e.* p-ACC (+35%; p<0.01; Suppl. Fig. 5A), fatty acid synthase (+101%;p<0.01; Suppl. Fig. 5B) and PGC1α (+27%; p<0.01; Suppl. Fig. 5C) *vs.* NTG mice. In the SOL, p-ACC and fatty acid synthase were not different between conditions, but PGC1α was lower expressed in ALS (-29%; p<0.01) (Suppl. Fig. 5A,B,C).

Finally, myogenic regulators were also differentially affected by ALS in the TA and SOL. In TA, ALS exhibited a lower expression of Myf5 (-159%; p<0.01; Suppl. Fig. 6B) and eMyHC (-138%; p<0.01; Suppl. Fig. 6D) compared to NTG, whereas myogenin was more expressed (+61%; p<0.01; Suppl. Fig. 6C) and Pax7 (Suppl. Fig. 6A) was not different between both conditions. In the SOL, a completely different myogenic expression pattern was observed, as Pax7 (-79%; p<0.01; Suppl. Fig. 6A) was lower expressed and myogenin (-36%; p=0.07; Suppl. Fig. 6C) tended to be lower expressed in ALS *vs.* NTG. Myf5 (Suppl. Fig. 6B) and eMyHC (Suppl. Fig. 6D) were not different between both conditions.

Together, key regulators of muscle health, including metabolic, myogenic and stress systems, were affected by ALS, and in parallel with muscle mass changes, the protein marker profile was more affected in the TA compared to the SOL.

***Supplementary*** ***figure 1****. The effect of hSOD1 genotype and age on autophagy markers in the tibialis anterior (TA) and soleus muscle (SOL). Autophagy markers include microtubule-associated proteins 1A/1B light chain 3B (cytosolic LC3b-I & autophagosomal LC3b-II; panel A,B,C), p62 (panel D) and Autophagy related 5-12 (ATG5-12; panel E). The individual (dots and triangles) and average (bar) values of non-transgenic control mice (NTG; dots) and hSOD1-transgene mice (ALS; triangles) are presented. Age 1, 2 and 3 refer to an age of 92 days, 113 days and 138 days, respectively. P values of two-way analysis of variance, and Bonferroni-corrected post-hoc analyses are presented (n=7-8/group).*

***Supplementary*** ***figure 2****. The effect of hSOD1 genotype and age on the ubiquitin proteasome markers muscle RING-finger protein-1 (MuRF1) and atrogin 1 in the tibialis anterior (TA) and soleus muscle (SOL). The individual (dots and triangles) and average (bar) values of non-transgenic control mice (NTG; dots) and hSOD1-transgene mice (ALS; triangles) are presented. Age 1, 2 and 3 refer to an age of 92 days, 113 days and 138 days, respectively. P values of two-way analysis of variance (n=7-8/group).*

***Supplementary*** ***figure 3****. The effect of hSOD1 genotype and age on anabolic markers in the tibialis anterior (TA) and soleus muscle (SOL). Anabolic markers include phosphorylated mammalian target of rapamycin (p-mTOR; panel A), phosphorylated S6 ribosomal protein (p-S6rp; panel B), phosphorylated Eukaryotic translation initiation factor 4E-binding protein 1 (p-4E-BP1; panel C) and phosphorylated eukaryotic translation elongation factor 2 (p-eEF2; panel D). The individual (dots and triangles) and average (bar) values of non-transgenic control mice (NTG; dots) and hSOD1-transgene mice (ALS; triangles) are presented. Age 1, 2 and 3 refer to an age of 92 days, 113 days and 138 days, respectively. P values of two-way analysis of variance, and Bonferroni-corrected post-hoc analyses are presented (n=7-8/group).*

***Supplementary*** ***figure 4****. The effect of hSOD1 genotype and age on inflammatory and stress markers in the tibialis anterior (TA) and soleus muscle (SOL). Inflammatory and stress markers include the macrophage marker F4/80 (panel A), the apoptotic markers caspase-3 (panel B) and Bax (panel C) and the endoplasmatic reticulum marker binding immunoglobulin protein (BiP; panel D). The individual (dots and triangles) and average (bar) values of non-transgenic control mice (NTG; dots) and hSOD1-transgene mice (ALS; triangles) are presented. Age 1, 2 and 3 refer to an age of 92 days, 113 days and 138 days, respectively. P values of two-way analysis of variance, and Bonferroni-corrected post-hoc analyses are presented (n=7-8/group except for n=6 for BiP of SOL in ALS age 2 and 3).*

***Supplementary*** ***figure 5****. The effect of hSOD1 genotype and age on metabolic markers in the tibialis anterior (TA) and soleus muscle (SOL). Metabolic markers include phosphorylated acetyl-CoA carboxylase (p-ACC; panel A), fatty acid synthase (panel B) and peroxisome proliferator-activated receptor-gamma coactivator 1α (PGC1α; panel C). The individual (dots and triangles) and average (bar) values of non-transgenic control mice (NTG; dots) and hSOD1-transgene mice (ALS; triangles) are presented. Age 1, 2 and 3 refer to an age of 92 days, 113 days and 138 days, respectively. P values of two-way analysis of variance, and Bonferroni-corrected post-hoc analyses are presented (n=7-8/group except for n=6 for p-ACC in ALS age 2).*

***Supplementary*** ***figure 6****. The effect of hSOD1 genotype and age on myogenic markers in the tibialis anterior (TA) and soleus muscle (SOL). Myogenic markers include paired box 7 (Pax7; panel A), myogenic factor 5 (Myf5; panel B), myogenin (panel C) and embryonal myosin heavy chain (eMyHC; panel D). The individual (dots and triangles) and average (bar) values of non-transgenic control mice (NTG; dots) and hSOD1-transgene mice (ALS; triangles) are presented. Age 1, 2 and 3 refer to an age of 92 days, 113 days and 138 days, respectively. P values of two-way analysis of variance, and Bonferroni-corrected post-hoc analyses are presented (n=7-8/group).*

***Supplementary figure 7****. The effect of hSOD1 genotype at the presymptomatic age of 56 days on the absolute weight of the tibialis anterior muscle (panel A), the extensor digitorum longus muscle (extensor DL; panel B), the soleus muscle (panel C) and the gastrocnemius muscle (panel D). The individual (dots and triangles) and average (bar) values of non-transgenic control mice (NTG; dots) and hSOD1-transgene mice (ALS; triangles) are presented. P values of unpaired T-test (normally distributed) or Mann-Whitney U test (non-normally distributed between both genotypes are presented (n=8/group).*

***Supplementary figure 8****. The effect of hSOD1 genotype at the presymptomatic age of 56 days on the protein expression of (p-mTOR; panel A), (p-S6rp; panel B), (p-4E-BP1; panel C), (p62; panel D), (LC3b-I; panel E), (LC3b-II; panel F), caspase-3 (panel G), myogenin (panel H), embryonal myosin heavy chain (eMyHC; panel I) in the tibialis anterior muscle compared to non-transgenic controls (NTG). The individual (dots and triangles) and average (bar) values of non-transgenic control mice (NTG; dots) and hSOD1-transgene mice (ALS; triangles) are presented. P values of unpaired T-test (normally distributed) or Mann-Whitney U test (non-normally distributed between both genotypes are presented (n=8/group).*

***Supplementary figure 9****. The effect of hSOD1 genotype at the presymptomatic age of 56 days on the expression of cannabinoid receptor 1 (CB1; panel A), diaglycerol lipase β (DAGLβ; panel B), monoacylglycerol lipase (MAGL; panel C), N-acyl phosphatidylethanolamine phospholipase D (NAPE-PLD; panel D) and fatty-acid amide hydrolase 1 (FAAH; panel E) in the tibialis anterior muscle compared to non-transgenic controls (NTG). The individual (dots and triangles) and average (bar) values of NTG (dots) and hSOD1-transgene mice (ALS; triangles) are presented. P values of* *unpaired T-test (normally distributed) or Mann-Whitney U test (non-normally distributed between both genotypes are presented (n=8/group).*

***Supplementary figure 10****. The effect of FAAH inhibition via URB937 treatment (1 mg/kg body weight intraperitoneally; 5x/week; n=9-10), compared to vehicle treatment (VEH; n=6), on the absolute weight of the tibialis anterior muscle (TA), the gastrocnemius muscle (GAS) and the soleus muscle (SOL). Difference in muscle mass was assessed via Student’s t-test. The individual (dots and rectangles) and average (bar) values of non-transgenic control mice (NTG; healthy reference group of 135 days old), and of VEH-treated and URB-treated hSOD1-transgene mice (ALS; triangles) are presented.*

***Supplementary*** ***figure 11****. The effect of daily URB937 (U; 1 mg/kg/d) vs. vehicle (V) treatment from the age of 80 days old until humane endpoints on the protein expression levels of markers of protein metabolism, muscle stress and neuromuscular junction in the tibialis anterior muscle of female hSOD193A. Tibialis anterior muscle samples from non-transgenic controls (C; 135 days old) were added as a healthy reference group. The vehicle- and URB937-treated groups were compared via the Student’s t-test. The individual (dots, squares and triangles) and average (bar) values are presented. P-mTOR: phosphorylated mechanistic target of rapamycin; CB1: cannabinoid receptor 1; LC3b: microtubule-associated protein 1 light chain 3 beta; F4/80: Bax: Bcl-2 Associated X-protein; MuSK: Muscle-Specific Kinase; NFH: neurofilament heavy chain; MuRF1: muscle-specific RING finger protein 1; PGC1α: peroxisome proliferator-activated receptor gamma coactivator 1-alpha (n=7-10/group).*

Principal component analyses of lipidomics in tibialis anterior muscle and soleus muscle of non-transgenic controls and hSOD1^93A^ mice

Tibialis anterior muscle

Soleus muscle
